# Scaffold-free human mesenchymal stem cell construct geometry regulates long bone regeneration

**DOI:** 10.1038/s42003-020-01576-y

**Published:** 2021-01-19

**Authors:** Samuel Herberg, Daniel Varghai, Daniel S. Alt, Phuong N. Dang, Honghyun Park, Yuxuan Cheng, Jung-Youn Shin, Anna D. Dikina, Joel D. Boerckel, Marsha W. Rolle, Eben Alsberg

**Affiliations:** 1 Department of Biomedical Engineering, Case Western Reserve University, 10900 Euclid Ave., Cleveland, OH 44106 USA; 2 Department of Aerospace and Mechanical Engineering, University of Notre Dame, Notre Dame, IN 46556 USA; 3 Department of Biomedical Engineering, Worcester Polytechnic Institute, 60 Prescott St, Worcester, MA 01605 USA; 4 Department of Orthopaedic Surgery, Case Western Reserve University, 2109 Adelbert Rd, Cleveland, OH 44106 USA; 5 National Center for Regenerative Medicine, Division of General Medical Sciences, Case Western Reserve University, 11100 Euclid Avenue, Cleveland, OH 44106 USA; 6 Present Address: Departments of Ophthalmology and Visual Sciences, Cell and Developmental Biology, and Biochemistry and Molecular Biology, SUNY Upstate Medical University, 505 Irving Ave, Syracuse, NY 13210 USA; 7 Present Address: Departments of Orthopaedic Surgery and Bioengineering, Perelman School of Medicine, University of Pennsylvania, 3400 Civic Center Blvd, Philadelphia, PA 19104 USA; 8 Present Address: Departments of Biomedical Engineering, Pharmacology, Orthopedics, and Mechanical & Industrial Engineering, University of Illinois, 909 S. Wolcott Avenue, Chicago, IL 60612 USA

**Keywords:** Stem-cell research, Regenerative medicine, Bone development

## Abstract

Biomimetic bone tissue engineering strategies partially recapitulate development. We recently showed functional restoration of femoral defects using scaffold-free human mesenchymal stem cell (hMSC) condensates featuring localized morphogen presentation with delayed in vivo mechanical loading. Possible effects of construct geometry on healing outcome remain unclear. Here, we hypothesized that localized presentation of transforming growth factor (TGF)-β1 and bone morphogenetic protein (BMP)-2 to engineered hMSC tubes mimicking femoral diaphyses induces endochondral ossification, and that TGF-β1 + BMP-2-presenting hMSC tubes enhance defect healing with delayed in vivo loading vs. loosely packed hMSC sheets. Localized morphogen presentation stimulated chondrogenic priming/endochondral differentiation in vitro. Subcutaneously, hMSC tubes formed cartilage templates that underwent bony remodeling. Orthotopically, hMSC tubes stimulated more robust endochondral defect healing vs. hMSC sheets. Tissue resembling normal growth plate was observed with negligible ectopic bone. This study demonstrates interactions between hMSC condensation geometry, morphogen bioavailability, and mechanical cues to recapitulate development for biomimetic bone tissue engineering.

## Abstract (web summary)

Herberg et al. previously showed functional healing of femoral defects using scaffold-free human mesenchymal stem cell (hMSC) condensates with localized morphogen presentation. In this study, they report the importance of the tubular geometry of MSC condensates in long bone regeneration. Unlike loosely packed hMSC sheets, only hMSC tubes induced regenerate tissue partially resembling normal growth plate.

## Introduction

Long bones of the appendicular skeleton such as the femur provide load-bearing structure and carry out critical metabolic functions^1^. Mesenchymal cell condensation in the early limb bud initiates long bone morphogenesis to form the cartilage anlage that gives rise to endochondral bone formation^2,3^. This process is dependent on local morphogen gradients and mechanical forces in utero (reviewed in ref. ^4^). Postnatal bone retains the remarkable ability to heal via endochondral ossification without fibrous scarring^5–7^; however, perturbations of the fracture site can interfere with the repair process when bone defects reach a critical size, which can contribute to non-union^8^.

Traditional scaffold-based bone tissue engineering approaches aim to create implantable constructs through a combination of biocompatible scaffolds, regeneration-competent cells, and/or bioactive cues^9^. Owing to the anatomy of the femoral diaphysis, studies have explored pre-formed tubular constructs in subcutaneous and orthotopic environments. Natural coral^10–12^ and synthetic polymers^13,14^ have been investigated as tubular scaffold materials for mesenchymal stem cells (MSCs). Furthermore, cell-free tubular composites of synthetic polymers and bone morphogenetic protein (BMP)-2 or BMP-7^15–18^ have been tested. Limitations of such strategies include (i) the use of scaffolds designed to match the properties of mature rather than developing tissues, (ii) the necessity to pre-culture MSCs for 2–3 weeks in an induction medium to stimulate osteogenic pre-differentiation, (iii) the delivery of a single morphogen, (iv) scaffold interference with critical cell–cell interactions, (v) scaffold degradation rate that is not in sync with new tissue formation, and (vi) potential immunogenicity or the cytotoxicity of the scaffold or its degradation byproducts.

Biomimetic bone tissue engineering approaches that recapitulate normal tissue development and remodeling have gained considerable interest as of late^19–21^. Scaffold-free cartilage templates derived from self-assembled human MSC (hMSC) condensations have been shown to progress through in vivo endochondral ossification, contingent on in vitro morphogen priming^22–28^. Limitations of this approach include (i) the necessity to pre-culture hMSC condensations for ≥3 weeks in an induction medium supplied with morphogens (e.g., transforming growth factor (TGF)-β1) to stimulate cartilage template formation, and (ii) and the inability to generate tubular tissue constructs. To that end, we recently reported a modular, scalable, scaffold-free system for engineering hMSC rings that can be assembled and fused into tubular structures^29–31^. The incorporation of TGF-β1-presenting gelatin microspheres for in situ chondrogenic priming^32^ and BMP-2-presenting mineral-coated hydroxyapatite microparticles to induce bony remodeling of the cartilaginous template^33^ circumvents the need for lengthy pre-differentiation and enables early in vivo implantation. Using this approach, we recently demonstrated functional femoral bone defect healing via endochondral ossification with microparticle-containing hMSC condensate sheets for a localized presentation of TGF-β1^34^ or TGF-β1 + BMP-2^35^ contingent on in vivo mechanical loading. Such high-density hMSC sheets are produced by seeding the cell-microparticle suspension atop standard Transwell membrane inserts. The cells coalesce through cell–cell interactions within 2 d of culture to yield a multi cell-layer condensate sheet with uniformly incorporated microparticles that can be lifted from the Transwell with ease and used for long bone defect healing applications. However, the effects of implant geometry—mimicking the geometry of long bone diaphyses with a channel for nutrient transport and endogenous cell invasion—on the healing outcome have not been assessed thus far.

In this study, we aimed to recapitulate the cellular, biochemical, and mechanical environment of the endochondral ossification process during early limb development for large long bone defect regeneration. We tested the hypotheses that (i) microparticle-mediated in situ presentation of TGF-β1 + BMP-2 to engineered hMSC condensate tubes induces the endochondral cascade in vitro and in a subcutaneous environment in vivo to a greater degree than the presentation of either morphogen alone, and that (ii) the geometry of TGF-β1 + BMP-2-presenting hMSC tubes, stabilized with custom compliant fixation plates that allow delayed initiation of in vivo loading, facilitate enhanced endochondral healing of critical-sized femoral segmental defects compared to loosely packed hMSC sheets with identical cell number and morphogen dosing.

## Results

### Overall study design

**Fig. 1:**
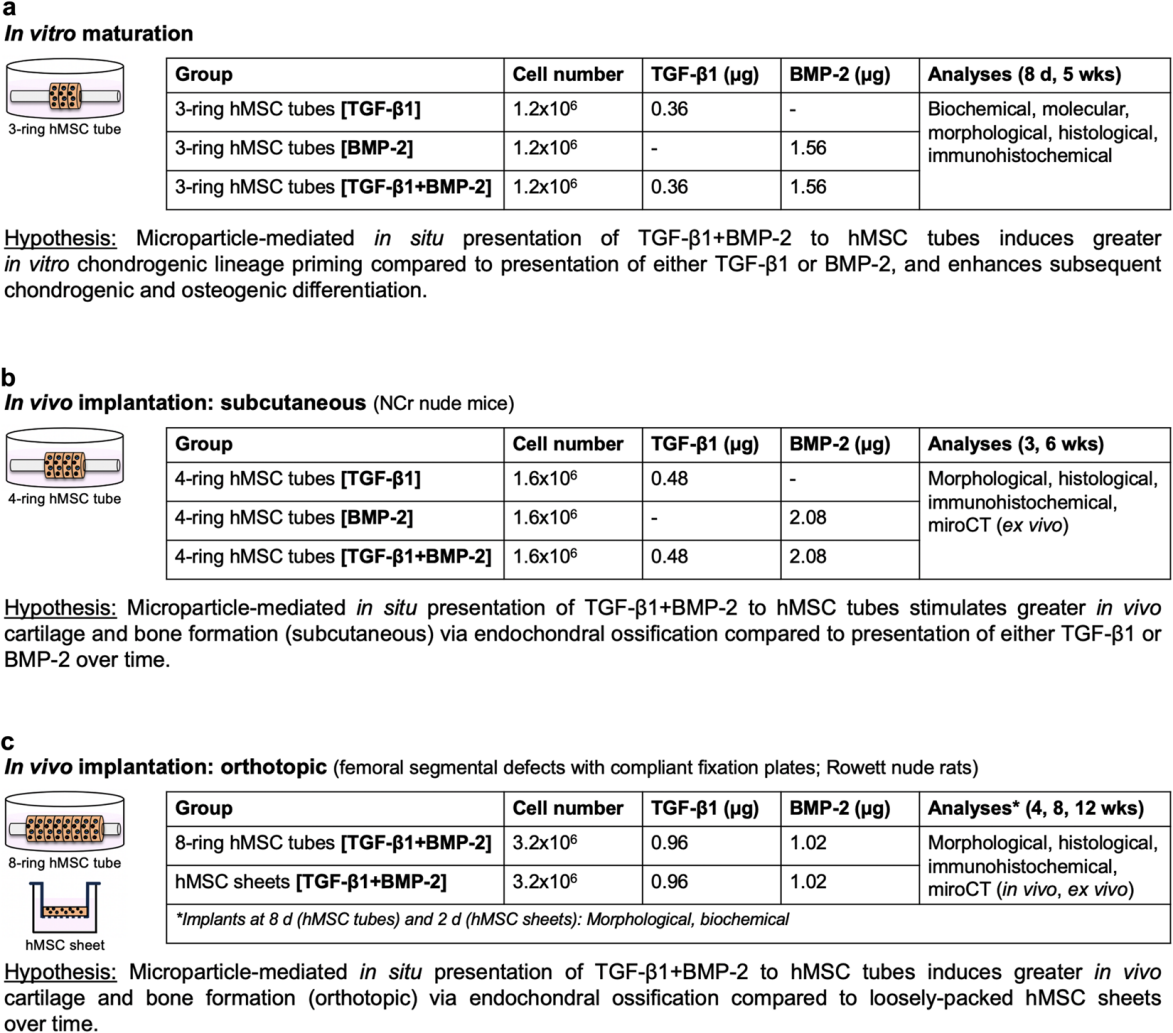
Study design and hypotheses. **a** In vitro maturation, **b** in vivo implantation (subcutaneous), and **c** in vivo implantation (orthotopic) of hMSC tubes containing morphogen-loaded microparticles.

The experimental design featured three parts: (a) in vitro maturation of 3-ring hMSC tubes containing microparticles to present TGF-β1, BMP-2, or TGF-β1 + BMP-2, (b) in vivo implantation of 4-ring hMSC tubes (subcutaneous; NCr-nude mice) containing microparticles to present TGF-β1, BMP-2, or TGF-β1 + BMP-2, and (c) in vivo implantation of 8-ring hMSC tubes or equivalent hMSC sheets containing microparticles to present TGF-β1 + BMP-2 (orthotopic with internal compliant fixation plates; Rowett nude rats) (Fig. 1).

### In vitro maturation of engineered tubular condensations

**Fig. 2:**
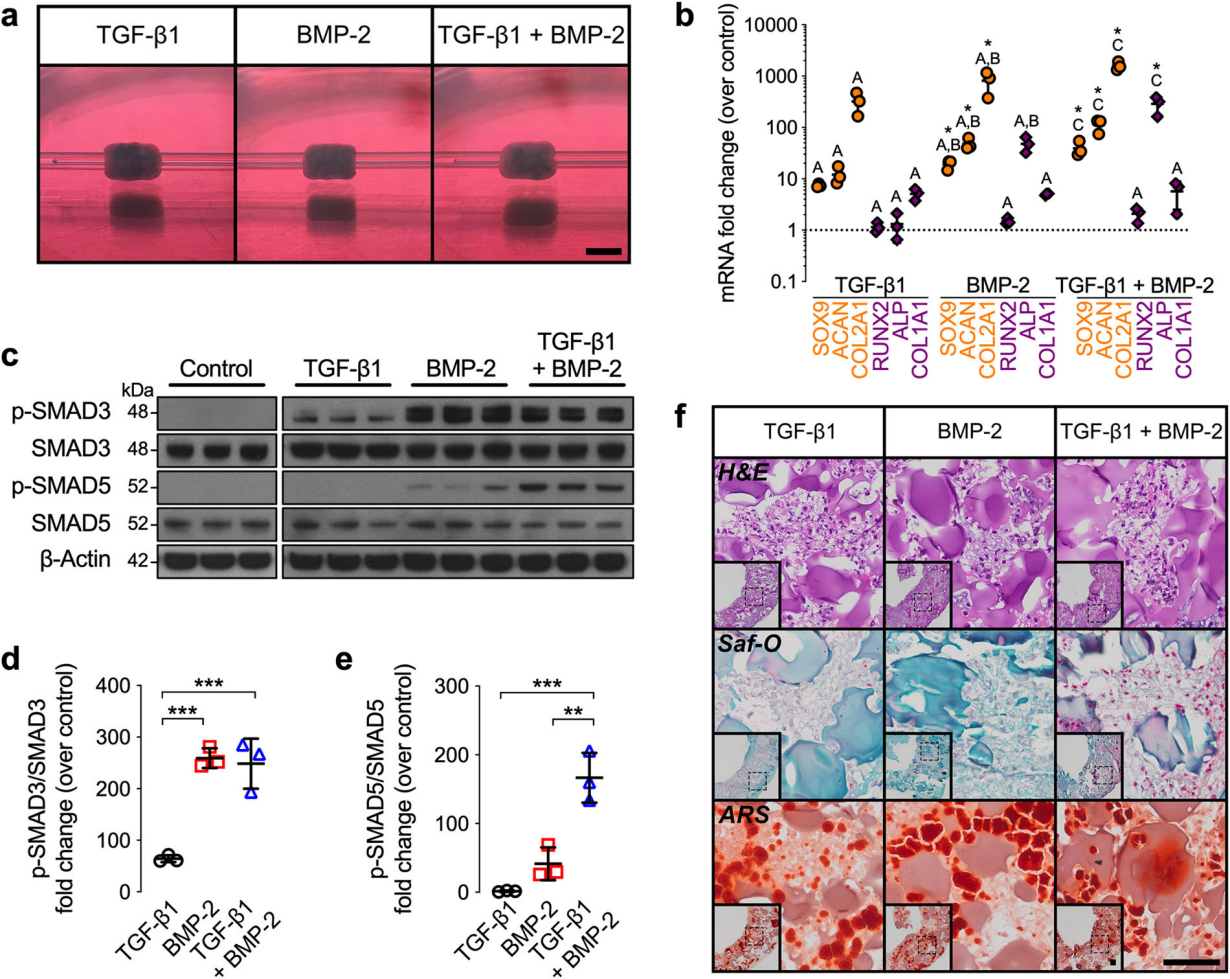
In vitro macroscopic, biochemical, molecular, and histological evaluation of engineered hMSC condensate tube early chondrogenic priming. **a** Representative gross macroscopic images of hMSC tubes containing TGF-β1-loaded, BMP-2-loaded, or TGF-β1 + BMP-2-loaded microparticles at day 8. Scale bar, 4 mm. **b** Normalized mRNA fold-change over control of key chondrogenic and osteogenic markers by qRT-PCR (*n* = 3 biologically independent samples per group; *p < 0.05 vs. control; orange circles = chondrogenic markers; purple diamonds = osteogenic markers). **c** Immunoblots and **d** relative fold-change over control of p-SMAD3/SMAD3, and **e** p-SMAD5/SMAD5. β-Actin served as a loading control (*n* = 3 biologically independent samples per group; **p < 0.01, ***p < 0.001; black circles = TGF-β1; red squares = BMP-2; blue triangles = TGF-β1 + BMP-2). **f** Representative histological Hematoxylin & Eosin (H&E), Safranin-O/Fast-green (Saf-O), and Alizarin Red S (ARS) staining of transaxial hMSC tube sections. Scale bars, 100 μm (dotted squares in insets show region of interest in high magnification image). Individual data points are shown with mean ± SD. Groups with shared letters have no significant differences. Analyzed by one-way ANOVA with Tukey’s post hoc test (*p* < 0.05 or lower considered significant).

Endochondral bone formation is initiated by condensation and chondrogenic lineage commitment of mesenchymal cells. Therefore, we seeded a suspension of hMSCs and microparticles to locally present (1) TGF-β1, (2) BMP-2, or (3) TGF-β1 + BMP-2 from within the maturing construct in custom agarose molds to facilitate tissue ring self-assembly by day 2, as described recently^30^. Tubular hMSC constructs were then created by placing several ring building blocks in direct contact with each other on horizontal glass tubes for an additional 6 d to facilitate tissue fusion (Supplementary Fig. S1 and Fig. 1a). We first investigated the effects of in situ morphogen presentation on hMSC tube lineage commitment at day 8 of culture. No gross morphological differences between groups were observed at this time point (Fig. 2a). Transcript analysis of key differentiation markers, normalized to controls without growth factors, revealed only minimally elevated mRNA expression of the chondrogenic genes sex-determining region Y-box 9 (SOX9), aggrecan (ACAN), and collagen type 2A1 (COL2A1), and the early osteogenic gene alkaline phosphatase (ALP) in BMP-2-loaded hMSC tubes compared to TGF-β1-presenting constructs (Fig. 2b). In contrast, TGF-β1 + BMP-2 dual presentation significantly potentiated SOX9, ACAN, COL2A1, and ALP mRNA levels compared to both TGF-β1- or BMP-2-only constructs (Fig. 2b). No differences in Runt-related transcription factor-2 (RUNX2) and collagen type 1A1 (COL1A1) expression were observed across groups (Fig. 2b). Immunoblot analysis showed SMAD3 phosphorylation in TGF-β1-loaded hMSC tubes; p-SMAD5 was not induced similar to controls without growth factor (Fig. 2c–e). In contrast, BMP-2 presentation induced significant SMAD3 phosphorylation and notable SMAD5 phosphorylation compared to TGF-β1-loaded hMSC tubes (Fig. 2c–e). While TGF-β1 + BMP-2 co-presentation did not further augment SMAD3 phosphorylation vs. BMP-2-loaded hMSC tubes, SMAD5 phosphorylation was significantly potentiated compared to either TGF-β1- or BMP-2-only constructs (Fig. 2c–e and Supplementary Fig. S10a). Histologically, engineered hMSC tubes displayed comparable 3-dimensional cellular organization across groups with relatively evenly distributed gelatin microspheres and no substantial glycosaminoglycan (GAG) or mineral deposition. Of note, the relatively proportionally dispersed Alizarin Red-staining observed in all groups was localized to incorporated mineral-coated hydroxyapatite microparticles (Fig. 2f and Supplementary Fig. S2a–c). Together, these findings suggest that while hMSC tubes across all groups were phenotypically undifferentiated at day 8 of culture, in situ presentation of TGF-β1, BMP-2, or TGF-β1 + BMP-2 imparted robust chondrogenic lineage priming in a morphogen-dependent manner.

**Fig. 3:**
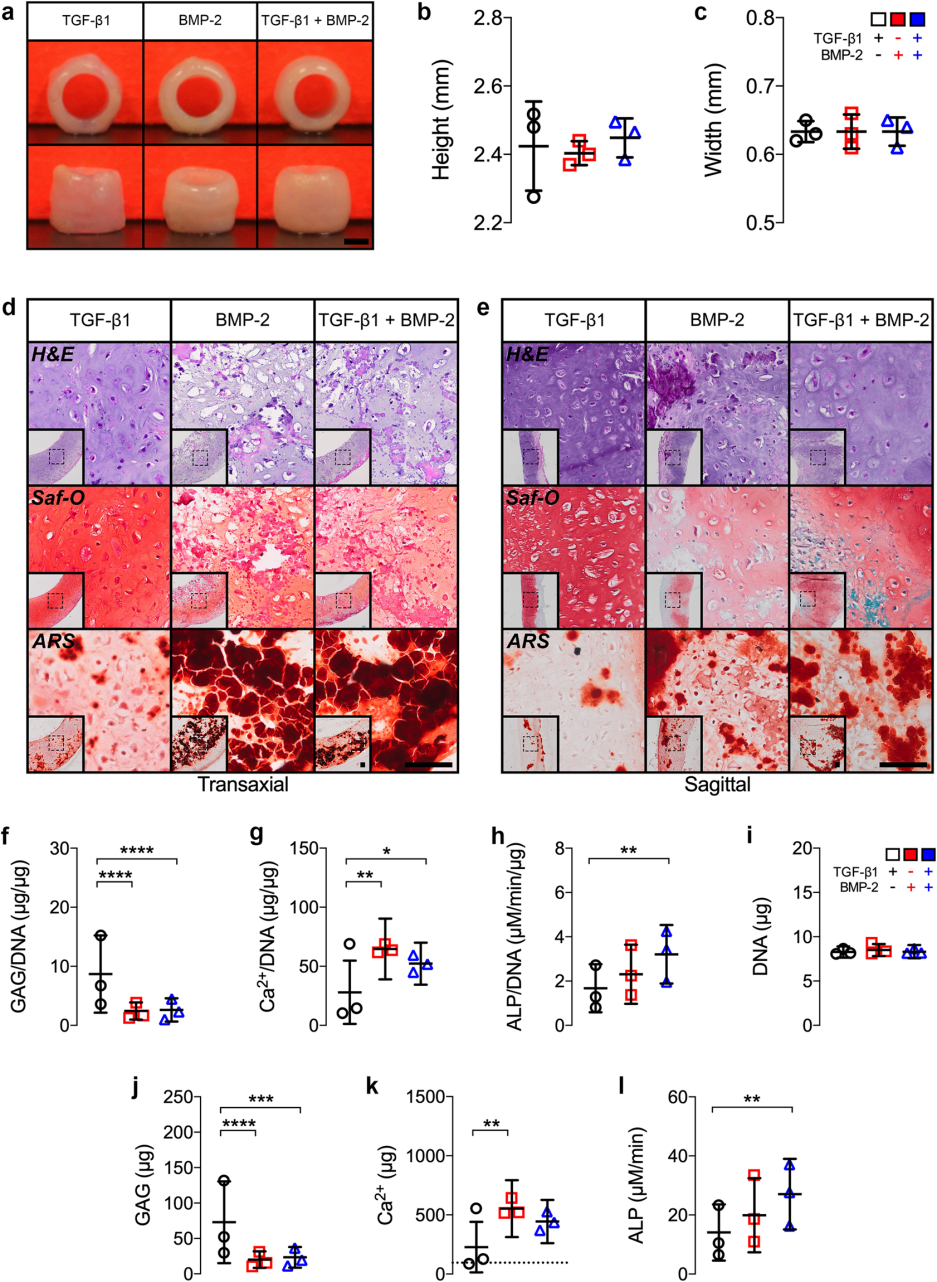
In vitro macroscopic, biochemical, and histological evaluation of engineered hMSC condensate tube maturation. **a** Representative gross macroscopic images of hMSC tubes containing TGF-β1-loaded, BMP-2-loaded, or TGF-β1 + BMP-2-loaded microparticles at week 5. Scale bar, 1 mm. **b**, **c** Quantification of hMSC tube wall width and height (*n* = 3 biologically independent samples per group; black circles = TGF-β1; red squares = BMP-2; blue triangles = TGF-β1 + BMP-2). **d**, **e** Representative histological Hematoxylin & Eosin (H&E), Safranin-O/Fast-green (Saf-O), and Alizarin Red S (ARS) staining of transaxial and sagittal hMSC tube sections. Scale bars, 100 μm (dotted squares in insets show region of interest in high magnification image). Quantification of **f** GAG/DNA content, **g** Ca^2+^/DNA content, **h** ALP activity/DNA, **i** DNA content, **j** GAG content, **k** Ca^2+^ content; dashed line represents the amount of Ca^2+^ initially contributed by mineral-coated hydroxyapatite microparticles assuming 100% incorporation, and **l** ALP activity (*n* = 3; derived from three hMSC donors (average of averages) each with 3 (TGF-β1; BMP-2) or 4 (TGF-β1 + BMP-2) biologically independent samples; *p < 0.05, **p < 0.01, ***p < 0.001, ****p < 0.0001; black circles = TGF-β1; red squares = BMP-2; blue triangles = TGF-β1 + BMP-2). Individual data points are shown with mean ± SD. Analyzed by one-way or two-way ANOVA with Tukey’s post hoc test (*p* < 0.05 or lower considered significant).

Next, we assessed the effects of microparticle-mediated TGF-β1, BMP-2, or TGF-β1 + BMP-2 presentation on stimulating hMSC tube differentiation. Constructs were cultured for 2 weeks in basal medium followed by 3 weeks in osteogenic medium (Supplementary Fig. S1 and Fig. 1a), conditions previously shown to facilitate robust chondrogenic and osteogenic fate specification in high-density hMSC constructs^30,36,37^. Gross morphological evaluation revealed comparable width and height of hMSC tubes across groups at 5 weeks (Fig. 3a–c). Histologically, engineered hMSC tubes displayed distinct patterns of tissue differentiation. While all constructs were relatively similar in size and cellularity across groups, presentation of TGF-β1 induced more robust and homogenous GAG deposition, exhibiting cells with chondrocyte-like morphology in Safranin-O-stained regions, compared to BMP-2- or TGF-β1 + BMP-2-loaded constructs (Fig. 3d, e and Supplementary Fig. S3m, n). Limited mineral deposition was observed with TGF-β1 presentation alone, similar to day 8 constructs. Conversely, BMP-2 and TGF-β1 + BMP-2 presentation induced substantial new mineral deposition by week 5 compared to TGF-β1-only hMSC tubes, consistent with the biochemical assessment (Fig. 3d, e and Supplementary Fig. S3m, n). Immunohistochemistry revealed robust Col II and Col X staining across groups, indicating the presence of both mature and hypertrophic chondrocytes. In contrast, Col I staining was only observed in BMP-2 and TGF-β1 + BMP-2-loaded hMSC tubes (Supplementary Fig. S4a–d). Tissue tubes using hMSCs derived from three different donors were prepared for biochemical analysis. The data showed significantly higher GAG/DNA and absolute GAG content with TGF-β1 presentation compared to BMP-2- or TGF-β1 + BMP-2-loaded constructs (Fig. 3f, j and Supplementary Fig. S3a, e, i). Conversely, BMP-2 and TGF-β1 + BMP-2 presentation promoted significantly higher Ca^2+^/DNA and BMP-2-only presentation promoted significantly greater total Ca^2+^ content relative to TGF-β1-only hMSC tubes (Fig. 3g, k and Supplementary Fig. S3b, f, j). ALP/DNA and absolute ALP activity were elevated with BMP-2 presentation, reaching significance with dual morphogen delivery vs. TGF-β1 (Fig. 3h, l and Supplementary Fig. S3c, g, k). No differences in DNA content were noted across groups (Fig. 3i and Supplementary Fig. S3d, h, l). Together, these findings suggest that in situ presentation of TGF-β1, BMP-2, or TGF-β1 + BMP-2 stimulated chondrogenesis, chondrocyte hypertrophy, and osteogenesis indicative of endochondral ossification of hMSC tubes by week 5 in vitro in a morphogen-dependent manner.

### In vivo performance of engineered tubular condensations—subcutaneous implantation

**Fig. 4:**
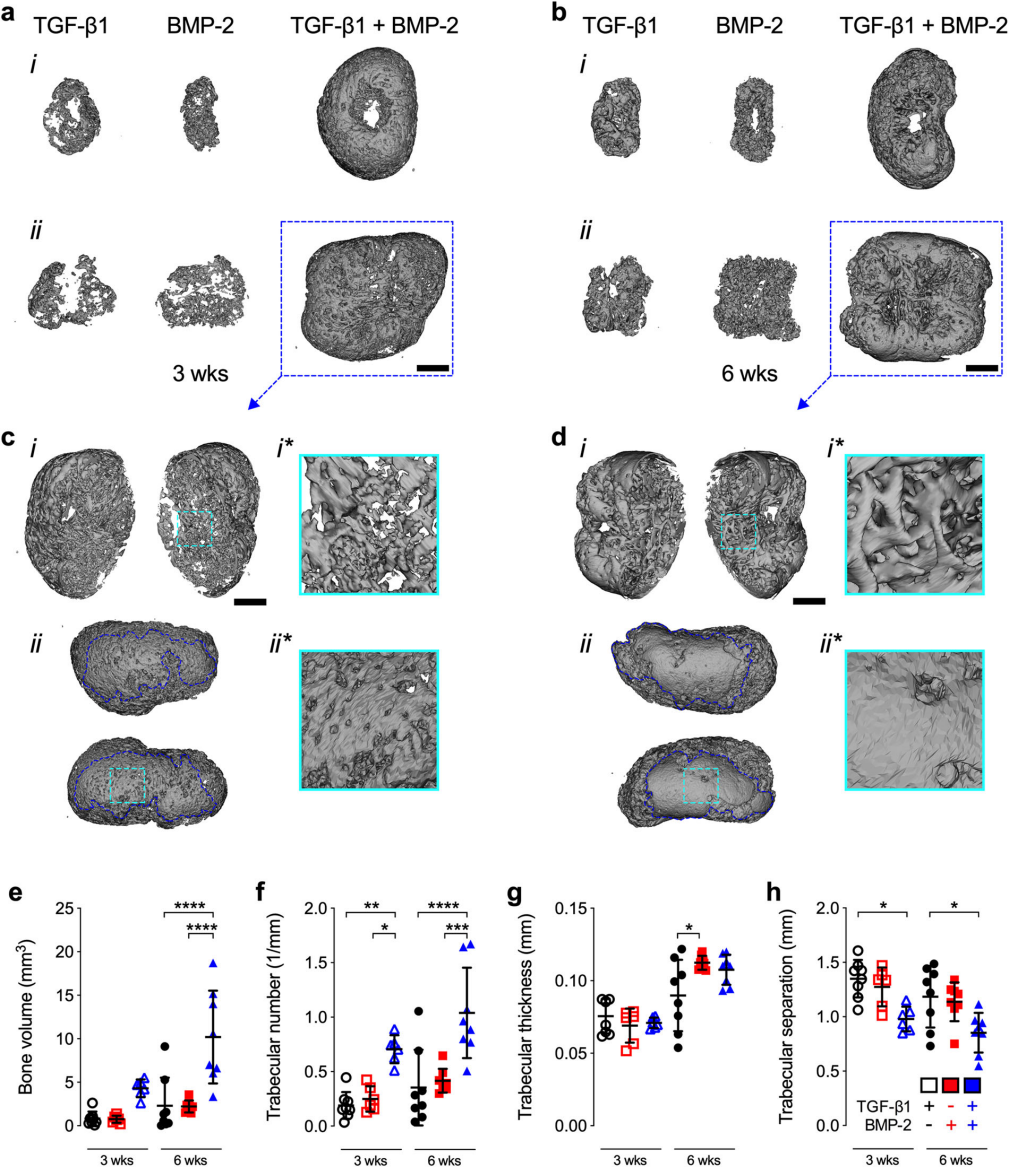
Ex vivo microCT evaluation of subcutaneous bone induced by engineered hMSC condensate tubes. **a**, **b** Representative 3-D microCT reconstructions of hMSC tube explants containing TGF-β1-loaded, BMP-2-loaded, or TGF-β1 + BMP-2-loaded microparticles at weeks 3 and 6, selected based on mean bone volume. Scale bars, 2 mm. (i) top view; (ii) side view. **c**, **d** Multiple angle views and close-ups of (i,i*) trabecular bone and (ii,ii*) cortical bone-like shell architecture in hMSC tube explants containing TGF-β1 + BMP-2-loaded microparticles (dotted blue squares in **a** and **b**) at weeks 3 and 6. Scale bars, 1 mm. Morphometric analysis of **e** bone volume, **f** trabecular number, **g** trabecular thickness, and **h** trabecular separation at weeks 3 and 6 (3 wks: *n* = 8 (TGF-β1) or 6 (BMP-2; TGF-β1 + BMP-2), and 6 wks: *n* = 8 (all groups) biologically independent samples per group; black circles = TGF-β1; red squares = BMP-2; blue triangles = TGF-β1 + BMP-2; open symbols = 3 wks; closed symbols = 6 wks). Individual data points are shown with mean ± SD. Analyzed by two-way ANOVA with Tukey’s post hoc test (*p* < 0.05 or lower considered significant).

To determine the ability of local TGF-β1, BMP-2, or TGF-β1 + BMP-2 presentation to modulate tissue formation in vivo, we subcutaneously implanted day 8 hMSC tubes in 9-week-old NCr-nude mice (Fig. 1b). Both TGF-β1- and BMP-2-presenting hMSC tube explants were significantly smaller at week 3 compared to TGF-β1 + BMP-2-loaded constructs, which had retained their average implant size; nearly identical patterns were observed at week 6 (Supplementary Fig. S5a–c). High-resolution ex vivo microCT analysis revealed limited ossification of TGF-β1- and BMP-2-presenting hMSC tubes at 3 and 6 weeks. Varying degrees of luminal collapse and substantial shrinkage were observed, consistent with the macroscopic evaluation. In contrast, TGF-β1 + BMP-2-loaded constructs underwent robust subcutaneous ossification at both time points with well-preserved luminal compartments and mineralization patterns guided by the overall implant geometry (Fig. 4a, bi, ii and Supplementary Movies S1, 2). Closer inspection of the trabecular architecture in the center of the TGF-β1 + BMP-2-loaded constructs at week 3 revealed relatively tightly packed, thin trabeculae (Fig. 4ci, i*). Four out of six hMSC tubes further exhibited condensed, shell-like mineralized structures on two opposing sides of the constructs’ outermost layer (Fig. 4cii, ii*). By week 6, central trabeculae appeared markedly thicker (Fig. 4di, i*). Eight out of eight hMSC tubes exhibited distinct dense outer mineral shells covering a significant portion of the tubes’ outer surface (Fig. 4dii, ii*). No such evidence was found in any of the TGF-β1- or BMP-2-presenting constructs at either time point. Morphometric analysis revealed markedly increased bone volume in TGF-β1 + BMP-2-loaded hMSC tubes at 3 weeks compared to TGF-β1- and BMP-2-only constructs, which did not differ from each other (Fig. 4e). By week 6, TGF-β1 + BMP-2-loaded tubes exhibited significantly greater bone volume relative to TGF-β1- and BMP-2-only constructs (Fig. 4e). Trabecular number was significantly increased in TGF-β1 + BMP-2-loaded tubes compared to TGF-β1- and BMP-2-only constructs at both time points (Fig. 4f). No differences in trabecular thickness were noted across groups at 3 weeks; however, both BMP-2- and TGF-β1 + BMP-2-loaded tubes exhibited markedly elevated trabecular thickness relative to TGF-β1-only constructs at 6 weeks, reaching significance for BMP-2 vs. TGF-β1 (Fig. 4g). Trabecular separation was markedly decreased in TGF-β1 + BMP-2-loaded tubes compared to TGF-β1- and BMP-2-only constructs at both time points, reaching significance vs. TGF-β1 (Fig. 4h).

**Fig. 5:**
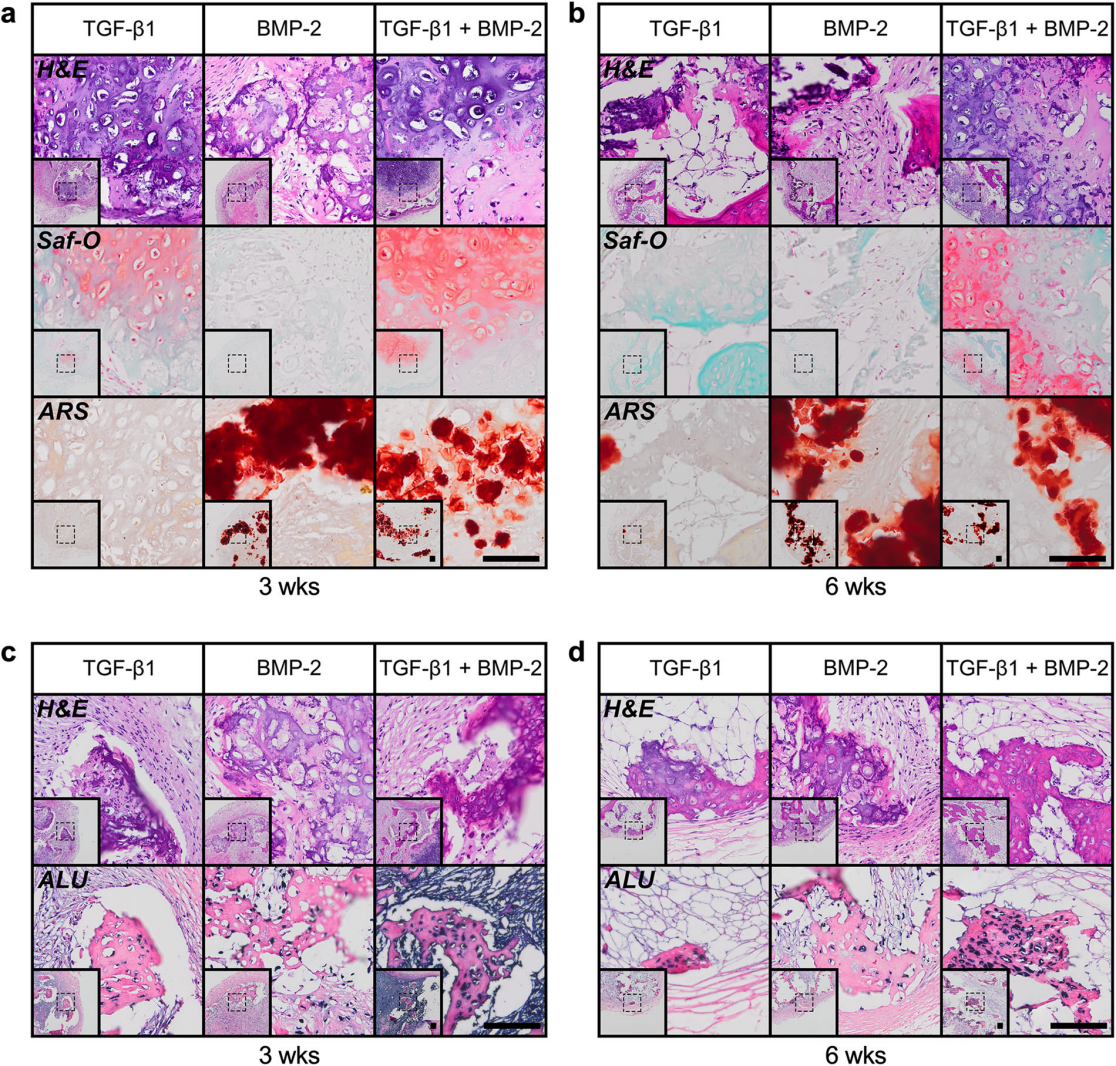
Ex vivo histological evaluation of subcutaneous bone tissue induced by engineered hMSC condensate tubes. **a**, **b** Representative histological Hematoxylin & Eosin (H&E), Safranin-O/Fast-green (Saf-O), and Alizarin Red S (ARS) staining of sagittal sections of hMSC tube explants containing TGF-β1-loaded, BMP-2-loaded, or TGF-β1 + BMP-2-loaded microparticles at weeks 3 and 6. **c**, **d** Representative H&E staining and in situ hybridization for human Alu repeats of transaxial hMSC tube explant sections at weeks 3 and 6. Scale bars, 100 μm (dotted squares in insets show region of interest in high magnification image).

Histologically, hMSC tube explants displayed distinct tissue patterns. TGF-β1-loaded constructs induced limited cartilage and bone formation at week 3 in vivo (Fig. 5a and Supplementary Fig. S5d). Presentation of BMP-2 exerted similar effects upon tube implantation; however, cartilage tissue was somewhat less pronounced whereas bone formation was more robust vs. TGF-β1-loaded constructs at week 3 (Fig. 5a and Supplementary Fig. S5d), consistent with the microCT evaluation. Strikingly, TGF-β1 + BMP-2-loaded hMSC tubes induced significant cartilage and bone formation guided by the implant geometry. Zonal cartilage containing both mature and hypertrophic chondrocytes with prominent GAG matrix were embedded in trabecular bone with osteoid occupying the transitional region, suggestive of endochondral ossification (Fig. 5a and Supplementary Fig. S5d). Nearly identical tissue formation patterns in a morphogen-dependent manner were observed at week 6 (Fig. 5b and Supplementary Fig. S5e). Across groups and time points, new tissue was largely comprised of human cells, as evidenced by in situ hybridization for human Alu repeats, while a mix of human and mouse cells contributed to the fibrous tissue infiltrating portions of the luminal space as well as surrounding the hMSC tube constructs (Fig. 5c, d). Immunohistochemistry corroborated the histological findings at 3 and 6 weeks. Col II and Col X staining as a proxy for identifying mature and hypertrophic chondrocytes, respectively, was most intense in areas with prominent GAG matrix, whereas Col I staining was strongest in regions with extensive mineral deposition (Supplementary Fig. S6a–d). Together, these findings suggest that localized presentation of TGF-β1, BMP-2, or TGF-β1 + BMP-2 stimulated in vivo cartilage and bone formation in hMSC tubes in a morphogen-dependent manner that was guided by the implant architecture. Importantly, TGF-β1 + BMP-2-loaded constructs formed a robust cartilaginous template that was actively remodeled into mineralized trabecular bone tissue through endochondral ossification in a subcutaneous environment. The presence of cortical bone-like compartments further suggests engagement of intramembranous ossification. Therefore, for subsequent studies, we focused on dual morphogen presentation.

### In vivo performance of engineered tubular condensations—orthotopic implantation

To evaluate the role of engineered condensation geometry on the induction of orthotopic bone regeneration, day-8 TGF-β1 + BMP-2-loaded hMSC tubes were implanted in critical-size femoral segmental defects in 12-week-old athymic nude rats (Fig. 1c). The hMSC tubes’ regenerative potential was compared to that of day-2 TGF-β1 + BMP-2-loaded hMSC sheets comprising matching cell numbers and morphogen-loaded microparticles. We previously reported successful calvarial^37^ and femoral segmental^35^ defect healing with dual morphogen-loaded hMSC sheets. For calvarial bone defects, the soft hMSC sheets can be implanted directly in the wound site without the need for additional supportive materials to keep the constructs in place. For femoral bone defects, however, the hMSC sheets are placed into thin, perforated, electrospun polycaprolactone (PCL) nanofiber mesh tubes^17,38,39^ (Supplementary Fig. S7a) to maintain construct shape and location and aid in handling/implantation, all while not hindering the constructs’ regenerative capacities. Femora were stabilized with custom fixation plates that permit dynamic tuning of plate compliance in vivo^34,35,38,40,41^. Similar to our prior studies, plates were initially implanted in a locked configuration (i.e., 0–4 weeks) for stable fixation, and then they were unlocked at week 4 to initiate ambulatory load transfer (i.e., 4–12 weeks) (Supplementary Fig. S7b), recently shown to enhance the restoration of bone function using growth factor-loaded hMSC sheets^34,35^.

Recognizing the differences in pre-culture times of the TGF-β1 + BMP-2-loaded condensations prior to implantation, 8 days for hMSC tubes and 2 days for hMSC sheets, we evaluated one randomly selected construct per group prior to implantation. Histologically, hMSC tubes (~550 µm wall thickness) appeared denser with noticeable GAG deposition in the outermost tissue layer, but cells did not display mature chondrocyte morphology (Supplementary Fig. S8a, b). In contrast, hMSC sheets (~250 µm thickness) were more loosely packed within the PCL nanofiber mesh tube and showed no evidence of GAG deposition. In both groups, ARS staining was comparable and limited to incorporated mineral-coated hydroxyapatite microparticles. In situ hybridization for human Alu repeats confirmed the cells’ identity (Supplementary Fig. S8a, b). Immunoblot analysis revealed robust SMAD3 and SMAD5 phosphorylation in both groups, with significantly higher levels in hMSC tubes vs. sheets (Supplementary Figs. S8c–e and S10b).

**Fig. 6:**
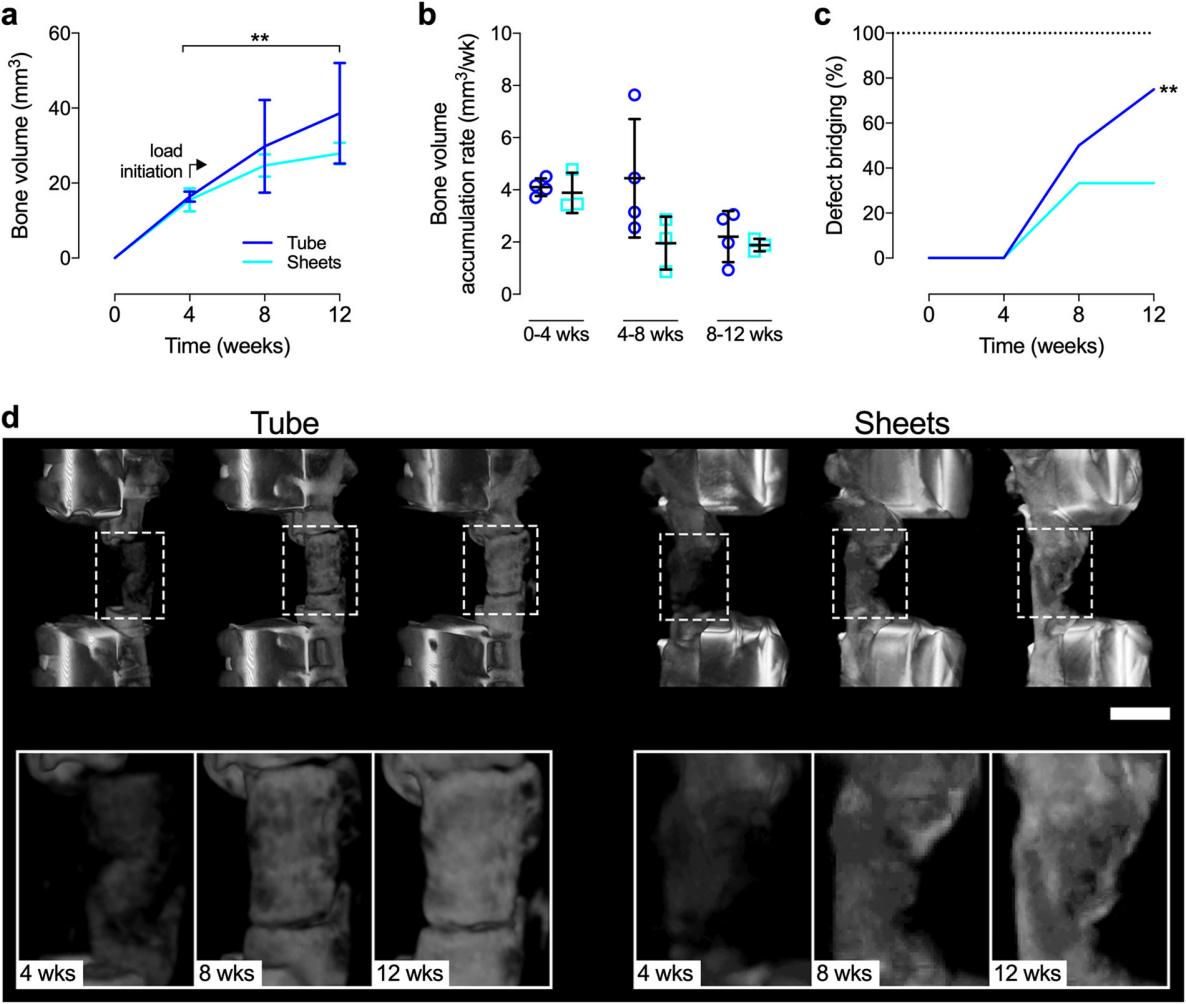
In vivo microCT evaluation of femoral defect healing induced by engineered hMSC condensation tubes and sheets. Longitudinal morphometric analysis of **a** bone volume and **b** bone volume accumulation rate at weeks 4, 8, and 12 in defects implanted with hMSC tubes or sheets containing TGF-β1 + BMP-2-loaded microparticles. Analyzed by two-way ANOVA with Tukey’s post hoc test (*p* < 0.05 or lower considered significant). **c** Longitudinal determination of defect bridging, defined as mineral fully traversing the defect (*n* = 4 (tubes) or 3 (sheets) biologically independent samples per group; blue lines/circles = hMSC tubes; cyan lines/squares = hMSC sheets). Significance of trend analyzed by chi-square test (**p < 0.01). **d** Representative 3-D microCT defect reconstructions selected based on mean bone volume. Scale bar, 5 mm. Individual data points are shown with mean ± SD.

Longitudinal in vivo microCT analysis showed increased bone formation with both TGF-β1 + BMP-2-loaded hMSC tubes and sheets upon implantation in femoral segmental defects over time. Bone volume levels at week 4 were comparable between groups; however, hMSC tubes continued to stimulate bone formation in a near-linear fashion upon load initiation at this time point through week 12, reaching significance compared to week 4 (Fig. 6a, d). In contrast, the rate of hMSC sheet-induced bone formation noticeably decreased after week 4 (Fig. 6a, d). Coinciding with plate unlocking, bone volume accumulation rate with hMSC tubes markedly increased between weeks 4 and 8; opposite trends were noted with hMSC sheets. By week 12, bone volume accumulation rates were comparable between groups (Fig. 6b). None of the defects achieved bridging, defined as mineralized tissue fully traversing the defect, at week 4. Fifty percent of defects implanted with TGF-β1 + BMP-2-loaded hMSC tubes were bridged by week 8, whereas hMSC sheets yielded only 33% bridging. By week 12, defect bridging reached 75% with hMSC tubes exhibiting an increasing trend over time, whereas levels remained unchanged with hMSC sheets (Fig. 6c).

**Fig. 7:**
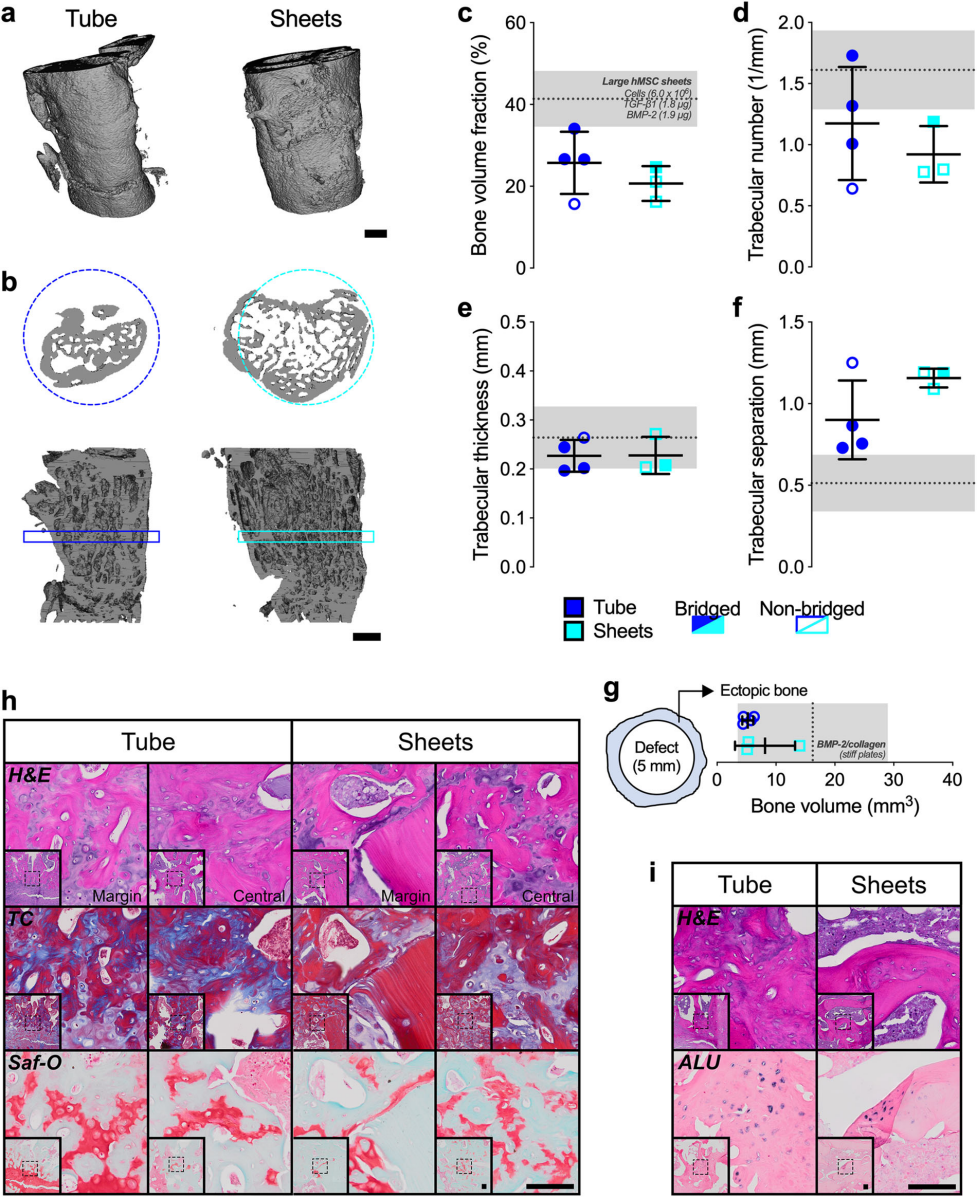
Ex vivo microCT and histological evaluation of femoral defect healing induced by engineered hMSC condensate tubes and sheets. **a** Representative 3-D microCT defect reconstructions, implanted with hMSC tubes or sheets containing TGF-β1 + BMP-2-loaded microparticles, with **b** mid-shaft transaxial (top) and sagittal (bottom) sections at week 12, selected based on mean bone volume. Scale bars, 2 mm. Morphometric analysis of **c** bone volume fraction, **d** trabecular number, **e** trabecular thickness, **f** trabecular separation, and **g** ectopic bone volume (i.e., bone extending beyond the 5-mm defect diameter) (*n* = 4 (tubes) or 3 (sheets) biologically independent samples per group; blue circles = hMSC tubes; cyan squares = hMSC sheets; open symbols = non-bridged; closed symbols = bridged) shown with reference data from large hMSC sheets containing TGF-β1 + BMP-2-loaded microparticles in **c**–**f** (from ref. ^33^), or BMP-2 soaked on collagen in **g** (from ref. ^33^). **h** Representative histological Hematoxylin & Eosin (H&E), Masson’s Trichrome (TC), and Safranin-O/Fast-green (Saf-O) staining of sagittal defect explant sections showing the defect margin and central defect; images oriented distal-to-proximal from bottom-to-top. **i** Representative H&E staining and in situ hybridization for human Alu repeats of sagittal defect explant sections. Scale bars, 100 μm (dotted squares in insets show region of interest in high magnification image). Individual data points are shown with mean ± SD. Analyzed by unpaired Student’s *t*-test (*p* < 0.05 or lower considered significant).

High-resolution ex vivo microCT analysis at 12 weeks demonstrated relatively uniform proximal-to-distal bone distribution in defects treated with either hMSC tubes or sheets with new bone largely confined to the 5-mm defect diameter (Fig. 7a, b and Supplementary Movie S3). TGF-β1 + BMP-2-loaded hMSC tubes induced a greater average bone volume fraction compared to hMSC sheets (Fig. 7c), consistent with the longitudinal in vivo assessment. Similarly, while not reaching significance, hMSC tube-treated defects displayed increased average trabecular number and connectivity density vs. hMSC sheets (Fig. 7d and Supplementary Fig. S9a). The expected inverse relationship was observed for trabecular separation, while trabecular thickness and degree of anisotropy were equivalent across groups (Fig. 7e, f and Supplementary Fig. S9b). MicroCT-based assessment of structural properties at 12 weeks showed enhanced mean and minimum polar moment of inertia (pMOI) with hMSC tubes compared to hMSC sheets (Supplementary Fig. S9c, d). Of note, both hMSC tubes and sheets induced negligible ectopic bone formation (i.e., bone extending beyond the 5-mm defect diameter; Fig. 7g), with overall levels markedly lower compared to those recently obtained with BMP-2 delivered on collagen^35^, suggesting a positive safety profile of BMP-2 delivered from scaffold-free, self-assembled hMSC condensations.

**Fig. 8:**
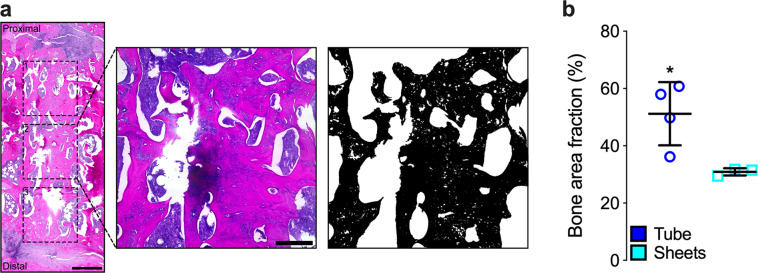
2-D bone histomorphometric evaluation of femoral defect healing induced by engineered hMSC condensate tubes and sheets. **a** Representative histological Hematoxylin & Eosin (H&E) staining of a sagittal defect explant section (hMSC tube group; image-oriented distal-to-proximal from bottom-to-top) showing three equal-sized regions of interest in dotted squares that were used to create thresholded black-and-white image masks, with bone tissue in black and all other tissue in white. **b** Histomorphometric analysis of bone area fraction (*n* = 4 (tubes) or 3 (sheets) biologically independent samples per group; an average of three regions of interest per sample; blue circles = hMSC tubes; cyan squares = hMSC sheets). Scale bars, 1 mm and 250 μm. Individual data points are shown with mean ± SD. Analyzed by unpaired Student’s *t*-test (*p* < 0.05 or lower considered significant).

To assess whether in situ presentation of TGF-β1 + BMP-2 to engineered hMSC constructs facilitated endochondral regeneration, we performed histological analyses of the defect tissue at week 12. Both hMSC tubes and sheets induced robust ossification; well-defined trabeculae containing lacunae-embedded osteocytes were found along with cartilaginous structures at different stages of remodeling (Fig. 7g and Supplementary Fig. S9e). Engineered hMSC tubes promoted the formation of growth plate-like, transverse cartilage bands on both the proximal and distal sides connecting the newly formed tissue with the intact bone ends. These remarkable structures exhibited zonal organization of both mature and hypertrophic chondrocytes and were aligned along the principal ambulatory load axis. A prominent GAG matrix was found embedded in trabecular bone with osteoid occupying the transitional region (Fig. 7g and Supplementary Fig. S9e), indicative of endochondral ossification. No such structures were evident in any of the hMSC sheet-implanted defects. Regenerate bone tissue in both groups at 12 weeks was composed of rat cells and, as demonstrated by in situ hybridization for human Alu repeats, remaining human cells (Fig. 7i). Immunohistochemistry indicated the most intense Col II and Col X staining for mature and hypertrophic chondrocytes, respectively, in areas exhibiting robust GAG matrix (Supplementary Fig. S9f). Lastly, quantitative 2-D histomorphometry of new bone tissue within the femoral defects revealed significantly greater bone area fraction with hMSC tubes compared to hMSC sheets (Fig. 8).

Together, these findings suggest that localized presentation of TGF-β1 + BMP-2 to hMSC condensations stimulated in vivo cartilage and bone formation in an orthotopic environment, independent of implant geometry. However, defined tubular hMSC condensations, exhibiting a greater degree of chondrogenic priming at the time of implantation, facilitated more robust healing compared to loosely packed hMSC sheets with bony bridging achieved in the majority of critical-size defects. New bone was formed through endochondral ossification in both groups; however, only hMSC tubes induced regenerate tissue that was remarkably similar to that observed with hMSC condensations containing 50% more cells and morphogens^35^ resembling the normal growth plate architecture.

## Discussion

In this study, we report the capacity of scaffold-free hMSC tubes to generate bone tissue in vivo through endochondral ossification after only 8 days of pre-culture necessary to allow for tube formation by fusion of individual hMSC rings, and the ability to heal critical-sized rat femoral segmental defects. We employed a biomimetic bone tissue engineering strategy^19^ to partially recreate the cellular, biochemical, and mechanical environment of the endochondral ossification process during long bone development. Specifically, we utilized (i) cellular self-assembly to form tubular mesenchymal condensations, (ii) microparticle-mediated TGF-β1 + BMP-2 presentation to activate specific morphogenetic pathways in situ, and (iii) delayed in vivo mechanical loading to augment defect healing. As such, our approach produced a self-organized tubular construct capable of autonomously progressing through formation, maturation, and differentiation into a structured tissue in a manner comparable to normal embryonic development, a concept referred to as developmental engineering^23,42^, while approximating the geometry of the adult femoral diaphysis with a channel for nutrient transport and endogenous cell invasion.

Following a similar biomimetic strategy, others have used scaffold-free, self-assembled hMSC condensations to form cartilage templates that can undergo hypertrophy and progress through endochondral ossification in vivo to form ossicles in a subcutaneous environment, but only after lengthy TGF-β-mediated chondrogenic priming in vitro^22–27^. The first study that investigated orthotopic implantation of scaffold-free hMSC condensations and showed successful healing of rat femoral segmental bone defects stabilized with internal fixation plates^28^ also required lengthy in vitro pre-differentiation. We recently demonstrated femoral bone defect healing via endochondral ossification using microparticle-containing hMSC condensate sheets for localized presentation of TGF-β1^34^ or TGF-β1 + BMP-2^35^ that was contingent on in vivo loading. No lengthy pre-differentiation of the cellular implants was required. Despite these efforts, bona fide tubular tissue constructs have not been developed using such protocols.

This is in contrast to multiple cell-based studies employing traditional bone tissue engineering approaches^9^ to form tubular constructs. Natural coral exoskeleton from *Porites* sp.^10–12^, and synthetic polymers such as poly lactic-*co*-glycolic acid (PLGA)^13^ or polycaprolactone (PCL)^14^ have been investigated as tubular scaffolds for MSCs. Coral/MSC tubes produced mineralized tissue with shape and structure comparable to native long bones in vivo in a subcutaneous environment^11–13^, and facilitated near complete healing of sheep metatarsal segmental bone defects stabilized with anchor plates^10^. Similarly, implantation of PLGA tubes combined with delayed, minimally-invasive percutaneous injection of MSCs stimulated bone regeneration of sheep tibial segmental defects stabilized with dynamic compression plates^14,43^. Although not without merit, these studies were hampered by (i) the use of scaffolds designed to match the properties of mature rather than developing tissues, and (ii) the need for lengthy in vitro osteogenic pre-differentiation of MSCs. The risk of poor vascular infiltration of the engineered constructs and subsequent compromised cell survival^44^, and differences between the bone repair approaches and normal developmental programs^45^ are potential limitations for broad clinical adaptation of such cell-based bone regeneration strategies.

Recently, we reported a modular, scalable, scaffold-free system for engineering hMSC rings that can be assembled and fused into tubular structures^29–31^. Here, we also employed gelatin microspheres^32^ for early TGF-β1 presentation^36^, and mineral-coated hydroxyapatite microparticles^33^ for sustained BMP-2 presentation^36^. This combination exerts potent in situ chondrogenic priming effects on early hMSC condensations, consistent with our recent study^30^. Importantly, this approach renders time- and cost-intensive pre-differentiation of the cellular constructs obsolete. Both TGF-β1 and BMP-2 have essential roles during embryonic skeletal development and postnatal bone homeostasis; TGF-β signaling is paramount during pre-cartilaginous mesenchymal cell condensation^46^, whereas BMP-2 acts upstream of the key transcription factors sex-determining region Y-box (SOX) 5, 6, and 9 during early chondrocyte differentiation^47,48^ and is a potent osteogenic growth factor. Here, we showed that localized presentation of TGF-β1 + BMP-2 stimulated cartilage and bone formation in hMSC tubes in vivo in a subcutaneous environment guided by the implant geometry. A robust cartilage template exhibiting zonal architecture with both mature and hypertrophic chondrocytes that was actively remodeled into trabecular bone through endochondral ossification was observed, and the presence of cortical bone-like structures indicated engagement of intramembranous ossification with spatial fidelity. Upon implantation in femoral segmental defects, stabilized with custom internal compliant fixation plates that allow for the delayed commencement of in vivo mechanical loading after 4 weeks of stable fixation^34,35,38,41^, engineered hMSC tubes induced more robust bone healing compared to loosely packed hMSC sheets with defect bridging achieved in the majority of critical-size defects. Using minimization of the Akaike’s information criterion^49^, we recently showed that defect bridging, minimum pMOI, and bone volume fraction can serve as accurate predictors of maximum torque and torsional stiffness^34,35^. While these biomechanical properties were not directly assessed here, our findings of hMSC tubes inducing ~2.3-fold greater defect bridging, ~3.0-fold greater minimum pMOI, and ~1.2-fold greater bone volume fraction compared to hMSC sheets are consistent with these prior observations and indicate likely enhanced biomechanical competency of experimental limbs at 12 weeks following hMSC tube implantation. New bone was formed through endochondral ossification in both groups; however, only hMSC tubes induced regenerate tissue that partially resembled the normal growth plate architecture. Failure of hMSC sheets in the present study to facilitate the formation of these structures upon implantation that were observed in recent related studies using larger hMSC sheets^34,35^ may be due to differences in cell number and morphogen dosing. Compared to these previous studies, here we implanted smaller constructs comprised of hMSC sheets that contained ~50% fewer cells (3.2 × 10^6^ vs. 6.0 × 10^6^) and were loaded with ~50% less morphogens (TGF-β1: 1.0 vs. 1.8 µg; BMP-2: 1.0 vs. 1.9 µg) to match the hMSC tube composition. Examining bone volume fraction as a key morphometric readout, hMSC sheets here exhibited ~2-fold lower values vs. the larger sheets reported recently^35^, while bone volume fraction levels with hMSC tubes were only ~1.6-fold lower. Together, this suggests that implant geometry can positively affect the healing outcome in this model; however, uncovering the exact contributions and interactions of cell number, morphogen dosing, and construct geometry will require further investigation.

A key consideration of implementing cell condensation technology whether in a tube or sheet geometry, is how the constructs are handled during both harvesting and implantation into defects. Much of the early work of cell sheet technology was done using poly(*N*-isopropylacrylamide) (PIPAAm), a thermoresponsive polymer that changes hydrophilicity in response to temperature changes allowing for the release of cell sheets cultured on it^50^. This thermoresponsive polymer allows for harvesting thin cell sheets (i.e., no more than a few cell layers thick) for utilization in tissue engineering applications. Other approaches have utilized cell sheets supported by biodegradable scaffolds for orthopedic and dental tissue engineering applications^51,52^. However, recent studies^30,34,35,37^ including the one described herein have shown that cell sheets made from hMSCs grown on Transwell membranes can be generated of sufficient thickness to be harvested by simply peeling them off of the membranes as early as 2 d after initial cell seeding, and then they can be manipulated individually. Similarly, hMSC condensate rings can be manually assembled into tubes after 2 d, and resulting tubes can be implanted without a supporting biomaterial, if desired.

Limitations to our orthotopic study include (i) the difference in pre-culture time between hMSC tubes and sheets, and (ii) the small number of femoral defect samples. Regarding the former, for consistency with previous in vivo studies^34,35,37^, we elected to implant day 2 hMSC sheets in comparison to day 8 hMSC tubes. In doing so, we were able to perform correlative analyses of hMSC sheet-induced defect healing across studies, and to provide context for interpreting the proof-of-principle results obtained with hMSC tubes. The two types of hMSC condensations used in the current femoral defect study were matched in cell number, microparticle concentration, and morphogen dosing; however, the additional 6 days in culture facilitated greater chondrogenic priming of hMSC tubes vs. sheets. No detailed assessment of ECM present in the cellular constructs at the time of implantation was performed beyond histological staining for GAGs. Biochemical analysis of hMSC tubes during the fusion process revealed no detectable amounts of GAG until 3 d after self-assembly (i.e., 5 d in culture)^30^. Therefore, we reasoned that the contribution of potential ECM contained within the hMSC condensations at implantation is likely minimal. However, the presence of live cells was recently demonstrated to be important for defect healing; we showed that devitalization of hMSC sheets by freeze–thaw cycling (which will leave any available ECM physically present/functional) substantially reduced bone formation compared to live-cell controls^34^. It is because of these previous findings that the primary focus of the study was to compare scaffold-free, self-assembled hMSC condensations of different geometries following thorough characterization of the hMSC tubes. It would be interesting to investigate to what degree the extra pre-culture time contributed to the enhanced regenerative effects in comparison to the implant geometry, possibly with an in vivo comparison of decellularized hMSC condensations to assess the relative contribution of ECM vs. cells.

Regarding the latter limitation, ten rats required euthanasia 24 h post-surgery; animals presented with marked gastric distention due to ingested hardwood bedding material. This unexpected pica behavior was traced back to buprenorphine administration, as reported by others^53,54^. Consequently, the sample size may have hindered the statistical differences observed between groups in the femoral defect experiment. Using all available specimens, quantitative bone histomorphometry revealed significantly greater defect healing with hMSC tubes vs. hMSC sheets. It would be worthwhile to expand upon the proof-of-principle findings described here in future studies, in particular, to further investigate the degree of restoration of limb biomechanical properties—considered the ultimate functional test. Furthermore, longer observation times beyond 12 weeks might help address whether tubular condensations are capable of facilitating complete cortical remodeling and re-establishment of a medullary canal.

In conclusion, we describe a biomimetic bone tissue engineering approach that recapitulates certain aspects of the normal endochondral cascade in the developing limb. Implantation of chondrogenically primed hMSC tubes, achieved through in situ morphogen presentation rather than lengthy pre-culture, in large long bone defects that will not heal if left untreated stimulated endochondral ossification for significant bone regeneration with delayed in vivo mechanical loading. The tubular hMSC system is of clinical relevance for long bone regeneration, and our findings advance the present understanding in the field of developmental engineering.

## Methods

### Experimental design

The study design featured three parts (Fig. 1): (a) in vitro maturation of 3-ring hMSC tubes, (b) in vivo implantation of 4-ring hMSC tubes (subcutaneous; NCr-nude mice), and (c) in vivo implantation of 8-ring hMSC tubes (orthotopic [femoral segmental defects]; Rowett nude rats). In vitro: First, three groups of 3-ring tissue hMSC condensate tubes (1.2 × 10^6^ cells) were investigated in vitro at 8 d and 5 weeks (Fig. 1a): (1) with transforming growth factor-β1 (TGF-β1)-loaded gelatin microspheres (GM) (0.36 µg) and unloaded mineral-coated hydroxyapatite microparticles (MCM) **[TGF-β1]**, (2) with unloaded GM and bone morphogenetic protein-2 (BMP-2)-loaded MCM (1.56 µg) **[BMP-2]**, or (3) with TGF-β1-loaded GM (0.36 µg) and BMP-2-loaded MCM (1.56 µg) **[TGF-β1** + **BMP-2]** (*N* = 3–5 per group and donor). Subcutaneous: Next, 4-ring hMSC condensate tubes (1.6 × 10^6^ cells) of the same three groups (1) with TGF-β1-loaded GM (0.48 µg) and unloaded MCM, (2) with unloaded GM and BMP-2-loaded MCM (2.08 µg), or (3) with TGF-β1-loaded GM (0.48 µg) and BMP-2-loaded MCM (2.08 µg)] were implanted subcutaneously to determine bone formation in male NCr-nude mice at 3 and 6 weeks in vivo (*N* = 6–8 per group) (Fig. 1b); two constructs from the same group were on the same side in each animal, which were evenly matched with other groups among animals for homogeneous pairing distribution. Orthotopic: Lastly, 8-ring hMSC condensate tubes (3.2 × 10^6^ cells) incorporated with TGF-β1-loaded GM (0.96 µg) and BMP-2-loaded MCM (1.02 µg), contained within an electrospun, perforated PCL nanofiber mesh tube, were implanted in femoral segmental defects in male Rowett nude rats to determine longitudinal bone formation over 12 weeks in vivo compared to randomly-oriented hMSC sheets at equivalent cell number, microparticle concentration, and growth factor doses (*N* = 3–4 per group) (Fig. 1c); treatment groups were randomly allocated. Limbs were stabilized with axially-compliant fixation plates initially implanted in a locked configuration to prevent loading (*k*_axial_ = 250 ± 35 N/mm), but after four weeks the plates were surgically unlocked to enable load transfer (*k*_axial_ = 8.0 ± 3.5 N/mm) (Supplementary Fig. S7)^34,35,38,40,41^.

### hMSC isolation and expansion

Human bone marrow-derived mesenchymal stromal/stem cells (hMSCs) were derived from the posterior iliac crest of three healthy donors (M/26, F/25, and M/49 years of age) under a protocol approved by the University Hospitals of Cleveland Institutional Review Board. Cells were isolated using a Percoll (Sigma-Aldrich, St. Louis, MO) density gradient and cultured in low-glucose Dulbecco’s modified Eagle’s medium (DMEM-LG; Sigma-Aldrich) containing 10% pre-screened fetal bovine serum (FBS; Sigma-Aldrich), and 1% penicillin/streptomycin (P/S; Fisher Scientific). The first media change washed away non-adherent cells, and adherent cells received fresh media supplemented with 10 ng/ml fibroblast growth factor-2 (FGF-2, R&D Systems, Minneapolis, MN)^55,56^.

### Gelatin microsphere synthesis and TGF-β1 loading

Gelatin microspheres (GM)^30,34–36,57,58^ were synthesized from 11.1% (w/v) gelatin type A (Sigma-Aldrich) using a water-in-oil single emulsion technique and crosslinked for 4 h with 1% (w/v) genipin (Wako USA, Richmond, VA). Hydrated GM were light blue in color and predominantly spherical in shape with an average diameter of 52.9 ± 40.2 µm and a crosslinking density of 25.5 ± 7.0%^30^. Growth factor-loaded microspheres were prepared by soaking crosslinked, UV-sterilized GM in an 80 μg/ml solution of rhTGF-β1 (Peprotech, Rocky Hill, NJ) in phosphate-buffered saline (PBS) for 2 h at 37 °C. Unloaded GM without growth factor were hydrated similarly using only PBS.

### Hydroxyapatite microparticle mineral coating and BMP-2 loading

Mineral-coated hydroxyapatite microparticles (MCM) were kindly provided by Dr. William L. Murphy (University of Wisconsin, Madison, WI). A preparation using low carbonate (4.2 mM NaHCO_3_) coating buffer and detailed characterization has been reported previously^33,36^. Lyophilized MCM from the same batch as used in our previous studies^30,35,36^, were loaded with a 100 µg/ml solution of rhBMP-2 (Dr. Walter Sebald, Department of Developmental Biology, University of Würzburg, Germany; 1.6 or 6.4 µg/mg) in PBS for 4 h at 37 °C. BMP-2-loaded MCM were then centrifuged at 800×*g* for 2 min and washed 2× with PBS. Unloaded MCM without growth factor were incubated with PBS only and treated similarly.

### Preparation of culture wells

Custom annular culture well molds (2 mm diameter posts; 3.75 mm wide trough) were 3D printed (Objet260 Connex; Stratasys, Eden Prairie, MN). Polydimethylsiloxane (PDMS; Sylgard 184, Dow Corning, Midland, MI) was cured in the printed molds and served as a negative for casting 2% w/v agarose culture wells (Denville Scientific Inc., Metuchen, NJ). Prior to cell seeding, culture wells were incubated over-night in serum-free, chemically defined basal medium comprised of high-glucose DMEM (Sigma-Aldrich) with 1% ITS^+^ Premix (Corning; Fisher Scientific), 1 mM sodium pyruvate (HyClone; Fisher Scientific), 100 μM non-essential amino acids (Lonza, Basel, Switzerland), 100 nM dexamethasone (MP Biomedicals, Solon, OH), 0.13 mM l-ascorbic acid-2-phosphate (Wako), and 1% P/S (Fisher Scientific)^29–31^.

### Preparation of microparticle-incorporated hMSC condensate tubes

Expanded hMSCs (4.0 × 10^5^ cells/construct; passage 4) were thoroughly mixed with TGF-β1-loaded GM (0.4 µg/mg; 0.3 mg/construct) and BMP-2-loaded MCM (1.6 or 6.4 µg/mg; 0.08 mg/construct) in basal medium. Fifty microliters of the suspension were seeded in the agarose culture wells^29–31^ and allowed to self-assemble for 2 days with feeding after 1 day (Supplementary Fig. S1). Subsequently, tissue rings were removed from the culture wells and transferred to 2 mm diameter borosilicate glass tubes (Adams & Chittenden Scientific Glass, Berkeley, CA) for fusion in 3-, 4-, or 8-ring hMSC condensate tubes. Tissue tubes were cultured horizontally on modified custom polycarbonate holders^29,30^ in 60 mm Petri dishes (Corning) containing 10 ml basal medium for 2 weeks, before switching to osteogenic medium for 3 weeks. This time course was previously shown to facilitate robust endochondral ossification^30,36^. Osteogenic medium was identical to basal medium but contained 0.17 mM l-ascorbic acid-2-phosphate and 5 mM β-glycerophosphate^30,36,37^. Respective media were replaced every 2 d. hMSC tubes incorporated with TGF-β1-loaded GM and unloaded MCM, or unloaded GM and BMP-2-loaded MCM were prepared and cultured in a similar fashion. Of note, hMSC condensate tubes incorporated with unloaded GM and unloaded MCM were prepared and served as controls for qRT-PCR and immunoblot analysis. For in vivo studies, tubular constructs (subcutaneous: 1.6 × 10^6^ cells; orthotopic: 3.2 × 10^6^ cells) were cultured for a total of 8 days in basal medium prior to implantation, previously shown to be sufficient for fusion into tubes^30^.

### Preparation of microparticle-incorporated hMSC condensate sheets

Expanded hMSCs (2.0 × 10^6^ or 0.6 × 10^5^ cells/construct; passage 4) were thoroughly mixed with TGF-β1-loaded GM (0.4 µg/mg; 1.5 mg/construct) and BMP-2-loaded MCM (1.6 µg/mg; 0.4 mg/construct) in basal medium. Five hundred or one hundred and fifty microliters of the suspension, respectively, were seeded onto pre-wetted membranes of Transwell inserts (3 µm pore size, 12 mm [2.0 × 10^6^ cells/construct] or 6.5 mm diameter [0.6 × 10^5^ cells/construct]; Corning) and allowed to self-assemble (i.e., coalesce through cell–cell interactions) for 2 days (Supplementary Fig. S7)^32,35,37^. After 1 day, the medium in the lower compartment was replaced with a fresh basal medium. After 2 days, a multi cell-layer condensate sheet with uniformly incorporated microparticles forms that can be easily lifted from the Transwell membrane; three hMSC condensate sheets (one 2.0 × 10^6^ cells/construct plus two 0.6 × 10^5^ cells/construct) were used for implantation for a total of 3.2 × 10^6^ cells.

### Gross morphological assessment

Gross macroscopic images of in vitro hMSC condensate tubes at 8 days (*N* = 3) and subcutaneous hMSC condensate tube explants at 3 and 6 weeks (*N* = 6–8) were taken with a smartphone camera (Apple Inc., Cupertino, CA) and calibrated using a metric ruler held within the frame of the image. ImageJ software (v1.53a, National Institutes of Health, Bethesda, MD) was used to assess the tissue area of the hMSC condensate tubes after initial calibration. Gross macroscopic images of in vitro hMSC tubes at 5 weeks (*N* = 3) were taken with a digital SLR camera (Canon, Melville, NY). Tube wall width and height measurements were obtained using calipers (Fowler High Precision, Newton, MA) at the 12, 4, and 8 o’clock positions^30^.

### Quantitative reverse transcription-polymerase chain reaction (qRT-PCR) analysis

Day 8 hMSC condensate tubes were homogenized in TRI Reagent (Sigma-Aldrich) for subsequent total RNA extraction and cDNA synthesis (iScript™ kit; Bio-Rad, Hercules, CA). One hundred nanograms of cDNA were amplified in duplicates in each 40-cycle reaction using a Mastercycler (Eppendorf, Hauppauge, NY) with annealing temperature set at 60 °C, SYBR® Premix Ex Taq™ II (Takara Bio Inc., Kusatsu, Shiga, Japan), and custom-designed qRT-PCR primers (Supplementary Table S1; Life Technologies, Grand Island, NY)^30,34,35,37^. Transcript levels were normalized to GAPDH and mRNA fold-change calculated relative to control hMSC condensate tubes without growth factor using the comparative *C*_T_ method^59^.

### Immunoblot analysis

Day 8 hMSC condensate tubes (*N* = 3) were homogenized in CelLytic™ MT lysis buffer (Sigma-Aldrich) supplemented with Halt™ protease and phosphatase inhibitor cocktail (Thermo Scientific). Equal amounts (15 µg) of protein lysates, determined by standard BCA protein assay kit (Pierce; ThermoFisher Scientific), were subjected to SDS-PAGE using 10% NuPAGE® Bis-Tris gels (Invitrogen; ThermoFisher Scientific) and transferred to 0.45 µm PVDF membranes (Millipore, Billerica, MA). Membranes were blocked with 5% bovine serum albumin (BSA) in standard TBST. The phosphorylation of intracellular SMAD3 and SMAD5 was detected using specific primary antibodies (anti-phospho-SMAD3 [ab52903] 1:2000; anti-phospho-SMAD5 [ab92698] 1:2000: Abcam, Cambridge, MA) followed by HRP-conjugated secondary antibodies (Jackson ImmunoResearch, West Grove, PA). Subsequently, the blots of phosphorylated SMADs were stripped (Western Blot Stripping Buffer, Pierce; ThermoFisher Scientific) and re-probed for the detection of the respective total protein (anti-SMAD3 [ab40854] 1:2000; anti-SMAD5 [ab40771] 1:2000: Abcam; anti-β-Actin [A1978]: Sigma-Aldrich 1:50,000) with respective HRP-conjugated secondary antibodies (Jackson ImmunoResearch)^34,35^. Bound antibodies were visualized with the ECL detection system (Pierce; ThermoFisher Scientific) on autoradiography film (ThermoFisher Scientific). The intensity of immunoreactive bands was quantified using ImageJ software.

### Biochemical analysis

Week 5 hMSC condensate tubes (*N* = 3–4 per donor) were homogenized in papain solution pH 7.4 (Sigma-Aldrich)^60^, and assayed for alkaline phosphatase activity (ALP assay kit; Sigma-Aldrich), DNA (PicoGreen kit; Invitrogen, Carlsbad, CA)^61^, glycosaminoglycan (GAG) (dimethylmethylene blue kit; Sigma-Aldrich)^62^, and calcium content (o-cresophthalein complexone assay kit; Pointe Scientific, Canton, MI)^30,36^.

### Histological and immunohistochemical analysis

Week 5 in vitro hMSC condensate tubes (*N* = 3), weeks 3 and 6 subcutaneous explants (N = 3), and day 8 hMSC condensate tubes and sheets (*N* = 1) were fixed in 10% neutral buffered formalin (NBF) for 24 h at 4 °C before switching to 70% ethanol. Femora at 12 weeks were fixed in 10% NBF for 72 h at 4 °C and then transferred to 0.25 M ethylenediaminetetraacetic acid (EDTA) pH 7.4 for 14 d at 4 °C under mild agitation on a rocker plate, with changes of the decalcification solution every 3–4 days. Following paraffin processing, 5 µm sections were cut using a microtome (Leica Microsystems Inc., Buffalo Grove, IL) and stained with Hematoxylin & Eosin (H&E), Safranin-O/Fast-green (Saf-O), Alizarin Red S (ARS), Masson’s Trichrome (TC), or picrosirius red (PSR; Polysciences, Inc., Warrington, PA). For constructs used as orthotopic implants within nanofiber mesh tubes, hMSC tube wall width/thickness at day 8 and hMSC sheet thickness at day 2 were determined on H&E-stained photomicrographs of transaxial and sagittal sections (*N* = 1; an average of 6 measurements per sample) using ImageJ software. In situ hybridization to detect human Alu repeats was performed using an ISH iVIEW Blue Plus Detection Kit (Ventana Medical Systems Inc., Tucson, AZ) and a BenchMark ULTRA automated IHC/ISH slide staining system (Ventana) according to the manufacturer’s instructions. Immunohistochemistry was performed to examine the presence of collagen (Col) types I, II, and X. All sections were deparaffinized and rehydrated with decreasing concentrations of ethanol, and endogenous peroxidase activity was quenched by submerging slides in 30% (v/v) hydrogen peroxide/methanol (1:9) for 10 min. For epitope retrieval, sections were digested with pronase (1 mg/ml; Sigma-Aldrich) in PBS at RT for 15 min. Rabbit anti-human Col I (ab138492) 1:2000, rabbit anti-human Col II (ab34712) 1:1000, and rabbit anti-human Col X ((ab58632) 1:200; all from Abcam, Cambridge, MA) were used as primary antibodies. Rabbit IgG (Vector Laboratories) served as a negative control. The Histostain-Plus Bulk kit (Invitrogen; ThermoFisher Scientific, Waltham, MA) with aminoethyl carbazole (Invitrogen) was utilized according to the manufacturer’s instructions with Fast-green counterstain. Slides were mounted with glycerol vinyl alcohol (Invitrogen), and light microscopy images, employing linearly polarized light for PSR-stained sections, were captured using an Olympus BX61VS microscope (Olympus, Center Valley, PA) with a Pike F-505 camera (Allied Vision Technologies, Stadtroda, Germany).

### Subcutaneous implantation—subcutaneous bone formation model

Nine-week-old male NCr-nude mice were obtained from the Athymic Animal and Xenograft Core Facility at Case Western Reserve University (CWRU). Animals were anesthetized using 2% isoflurane. Small skin incisions were made on the dorsal side ~15 mm from the midline. Subcutaneous pockets were generated using blunt dissection and 4 hMSC condensate tubes (2 per side) were implanted per mouse approximately 30 mm from each other, cranial to caudal. Two constructs from the same group were on the same side in each animal, which were evenly matched with other groups among animals for homogenous pairing distribution between TGF-β1—BMP-2, TGF-β1—BMP-2/TGF-β1, and BMP-2—BMP-2/TGF-β1. The incisions were closed and animals were given subcutaneous injections of 0.1 mg/kg buprenorphine at 0 and 12 h postoperative (post-op). All procedures were performed in strict accordance with the NIH Guide for the Care and Use of Laboratory Animals, and the policies of the CWRU Institutional Animal Care and Use Committee (IACUC) (Protocol No. 2014-0096). Animals were euthanized at 3 and 6 weeks by CO_2_ asphyxiation followed by cervical dislocation. Skin flaps were opened and hMSC condensate tube explants retrieved.

### Nanofiber mesh production

Nanofiber meshes were formed by dissolving 12% (w/v) poly-(ε-caprolactone) (PCL; Sigma-Aldrich) in 90/10 (v/v) hexafluoro-2-propanol/dimethylformamide (Sigma-Aldrich). The solution was electrospun at a rate of 0.75 ml/h onto a static aluminum collector. 9 mm × 20 mm sheets were cut from the product, perforated with a 1 mm biopsy punch (VWR, Radnor, PA) (Supplementary Fig. S7), and glued into tubes around a 4.5 mm mandrel with UV glue (Dymax, Torrington, CT). Perforated PCL nanofiber mesh tubes were sterilized by 100% ethanol evaporation under UV light over-night and washed 3× with sterile PBS before use. One day 8, microparticle-incorporated hMSC condensate tube or three day 2 hMSC condensate sheets combined, each comprised of a total of 3.2 × 10^6^ cells, were added into a sterile perforated PCL mesh tube for orthotopic implantation.

### Orthotopic implantation—femoral segmental defect model

Critical-sized (8 mm) bilateral segmental defects were created in the femora of 12-week-old male Rowett nude (RNU) rats (Taconic Biosciences Inc., Hudson, NY) using an oscillating saw under isoflurane anesthesia^34,35,38,40,41,63^. Animals received subcutaneous injections of 4 mg/kg lidocaine with 2 mg/kg bupivacaine for local block. Anterolateral incisions were made over the length of each limb, and the *vastus intermedius* and *vastus lateralis* muscles were blunt-dissected to expose the femur. Limbs were stabilized by custom internal fixation plates that allow controlled transfer of ambulatory loads in vivo (Supplementary Fig. S7)^34,35,38,40,41,63^ and secured to the femur by four bi-cortical miniature screws (J.I. Morris Co, Southbridge, MA). Animals were given subcutaneous injections of 0.04 mg/kg buprenorphine every 8 h for the first 24 h post-op with 4 mg/kg carprofen every 24 h for 72 h. In addition, 5 ml of 0.9% NaCl were administered subcutaneously to aid in recovery. All procedures were performed in strict accordance with the NIH Guide for the Care and Use of Laboratory Animals, and the policies of the CWRU IACUC (Protocol No. 2015-0081). Animals were euthanized at 12 weeks by CO_2_ asphyxiation followed by cervical dislocation. Hind limbs were excised and femora retrieved.

### In vivo microCT

In vivo microcomputed tomography (microCT) scans were obtained in RNU rats at 4, 8, and 12 weeks to assess longitudinal femoral defect healing^34,35^. Data were acquired using an Inveon microPET/CT system (Siemens Medical Solutions, Malvern, PA) at 45 kVp, 0.2 mA, and 35 μm isotropic voxels, and reconstructed using system-default parameters for analyzing bone and accounting for the metal in the fixation plates. DICOM-exported files were processed for 3-D analysis (CTAn software, Skyscan; Bruker, Billerica, MA) using a gauss filter at 1.0-pixel radius and a global threshold range of 28–255 for all samples. This segmentation approach allowed viewing of the normal bone architecture in the binary images as seen in the original reconstructed images^64^. One hundred and eighty slices in the center of each defect were analyzed using a 10 mm diameter circle centered on the medullary canal to assess bone volume. A binary bridging score was assigned by two independent, blinded observers, and determined as mineralized tissue fully traversing the defect. Representative 3D images were created using Dornheim Segmenter software (Dornheim Medical Images, Magdeburg, Germany), selected based on average bone volume values.

### Ex vivo microCT

Ex vivo microCT scans were obtained at 3 and 6 weeks (mouse; subcutaneous model) or at 4, 8, and 12 weeks (rat; orthotopic model). Data were acquired using a Skyscan 1172 microCT scanner (Skyscan; Bruker) with a 0.5 mm aluminum filter at 75 kVp and 0.1 mA. Subcutaneous explants wrapped in gauze were placed in a plastic sample holder and scanned in PBS at 10 µm isotropic voxels, 1170 ms integration time, rotation step of 0.5°, and frame averaging of 5. All subcutaneous explants were scanned within the same container using the same scanning parameters. Femora wrapped in gauze were placed in a plastic sample holder with the long axis oriented parallel to the image plane and scanned in PBS at 20 µm isotropic voxels, 330 ms integration time, rotation step of 0.5°, and frame averaging of 5. All femora were scanned within the same container using the same scanning parameters. All scans were then reconstructed using NRecon software (Skyscan; Bruker) with the same reconstruction parameters (ring artifact reduction of 5, beam hardening correction of 20%). For 3-D analysis (CTAn software, Skyscan; Bruker), a gauss filter at 1.0-pixel radius and a global threshold range of 65–255 were used for all samples. This segmentation approach allowed viewing of the normal bone architecture in the binary images as seen in the original reconstructed images^64^. For subcutaneous explants, varying numbers of slices were analyzed in an 8 mm diameter circle to account for the differences in size. For femora, 325 slices in the center of each defect were analyzed using a 10 mm (total) or 5 mm (defect) diameter circle centered on the medullary canal. Bone volume, bone volume fraction, polar moment of inertia, and the morphometric parameters trabecular number, trabecular thickness, trabecular separation, degree of anisotropy, and connectivity density were calculated^64^. Representative 3D images were created using CTAn software (Skyscan), selected based on average bone volume values.

### Bone histomorphometry

The standard two-dimensional (2-D) histomorphometric parameter^65,66^ bone area fraction, which by definition is numerically identical with the corresponding bone volume fraction, was assessed in the center of each femoral defect (H&E-stained sagittal sections, three equal-sized square regions of interest spanning the length of the defect; Fig. 8a) according to established protocols^67–69^. First, black-and-white thresholded image masks of the H&E-stained color images were created using the wand tool (tolerance: 45) in Photoshop CC 2018 software v19.1.3 (Adobe Systems, San Jose, CA) to highlight areas of bone. Bone tissue was designated in black, while the remaining tissue was designated in white, creating the mask for analysis (Fig. 8a). Next, using ImageJ software, the entire image area was calculated as tissue area and, using the default wand tool, the combined black-colored areas were summarized as bone area to calculate bone area fraction (i.e., an average of three regions of interest per sample before an average of all samples per group).

### Statistics and reproducibility

Fold-changes over control in mRNA expression and ratios of phosphorylated SMADs/total SMADs, and differences in gross morphological hMSC condensate tube wall width and height, and biochemical markers were analyzed by Student’s unpaired *t*-test, one-way or two-way analysis of variance (ANOVA) with Tukey’s multiple comparison post hoc test, as appropriate. Differences in gross morphological tissue area, and ex vivo microCT bone volume, and 3-D morphometry in hMSC condensate tube explants were analyzed by two-way ANOVA with Tukey’s multiple comparison post hoc test. Bridging of femoral defects was determined by chi-square test for trend for each group. Differences in longitudinal in vivo microCT bone volume and bone volume accumulation rate at 4, 8, and 12 weeks were assessed by two-way ANOVA with Tukey’s multiple comparison post hoc test. Ex vivo microCT bone volume fraction and 3-D morphometry, and 2-D bone histomorphometry in femoral defects at 12 weeks were determined by Student’s unpaired *t*-test. Sample sizes were chosen based on previous studies from our group. No data were excluded from the analyses. Investigators were blinded to group allocation during data collection and analyses. All data are shown with mean ± SD, some with individual data points. The significance level was set at *p* < 0.05 or lower. Groups with shared letters have no significant differences. GraphPad Prism software v6.0 (GraphPad Software, La Jolla, CA) was used for all analyses.

### Reporting summary

Further information on research design is available in the Nature Research Reporting Summary linked to this article.

## Supplementary information

Supplemental Information

Description of Additional Supplementary Files

Supplementary Video 1

Supplementary Video 2

Supplementary Video 3

Supplementary Data 1

Reporting Summary

## Data Availability

All data needed to evaluate the conclusions in the paper are present in the paper and/or the Supplementary Information. Source data is available in Supplementary Data 1. Additional data related to this paper may be requested from the corresponding author (E.A.).
