# Supplementary material for: Scaffold-free human mesenchymal stem cell construct geometry regulates long bone regeneration
Source: Commun Biol. 2021 Jan 19;4:89. doi: 10.1038/s42003-020-01576-y (PMC7815708; doi:10.1038/s42003-020-01576-y)
Supplement: Supplementary file 1 — Supplemental Information [file 42003_2020_1576_MOESM1_ESM.pdf]

Supplementary Information

Table S1. Oligonucleotide primer sequences for qRT-PCR.

**Gene**	**Sequence (5'-3')**		**Accession number**
SOX9	Fwd	CACACAGCTCACTCGACCTTG	NM_000346.3
	Rev	TTCGGTTATTTTTAGGATCATCTCG	
ACAN	Fwd	TGCGGGTCAACAGTGCCTATC	NM_001135.3
	Rev	CACGATGCCTTTCACCACGAC	
COL2A1	Fwd	GGAAACTTTGCTGCCCAGATG	NM_001844.4
	Rev	TCACCAGGTTCACCAGGATTGC	
RUNX2	Fwd	ACAGAACCACAAGTGCGGTGCAA	NM_001015051.3
	Rev	TGGCTGGTAGTGACCTGCGGA	
ALP	Fwd	CCACGTCTTCACATTTGGTG	NM_000478.4
	Rev	GCAGTGAAGGGCTTCTTGTC	
COL1A1	Fwd	GATGGATTCCAGTTCGAGTATG	NM_000088.3
	Rev	GTTTGGGTTGCTTGTCTGTTTG	
GAPDH	Fwd	GGGGCTGGCATTGCCCTCAA	NM_002046.5
	Rev	GGCTGGTGGTCCAGGGGTCT	

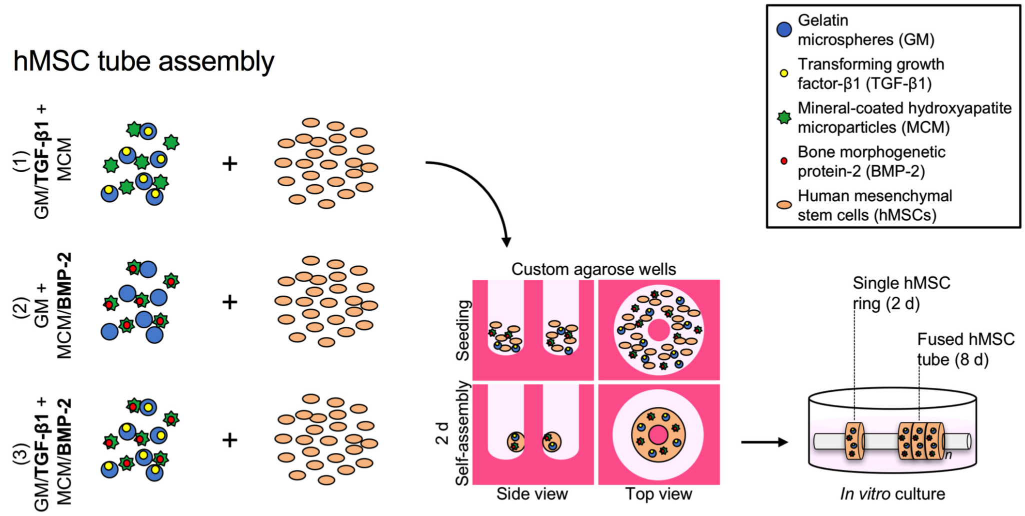

Fig. S1. Schematic of hMSC condensate tube assembly and culture. hMSCs were mixed with (1) TGF-β1-loaded gelatin microspheres and unloaded mineral-coated hydroxyapatite microparticles [TGF-β1], (2) unloaded gelatin microspheres and BMP-2-loaded mineral-coated hydroxyapatite microparticles [BMP-2], or (3) TGF-β1-loaded gelatin microspheres and BMP-2-loaded mineral-coated hydroxyapatite microparticles [TGF-β1 + BMP-2], seeded in custom agarose culture wells, and allowed to self-assemble into hMSC rings for 2 days before fusion into tubes by 8 days. hMSC tubes were cultured horizontally on glass tubes for 2 weeks in basal medium followed by 3 weeks in osteogenic medium.

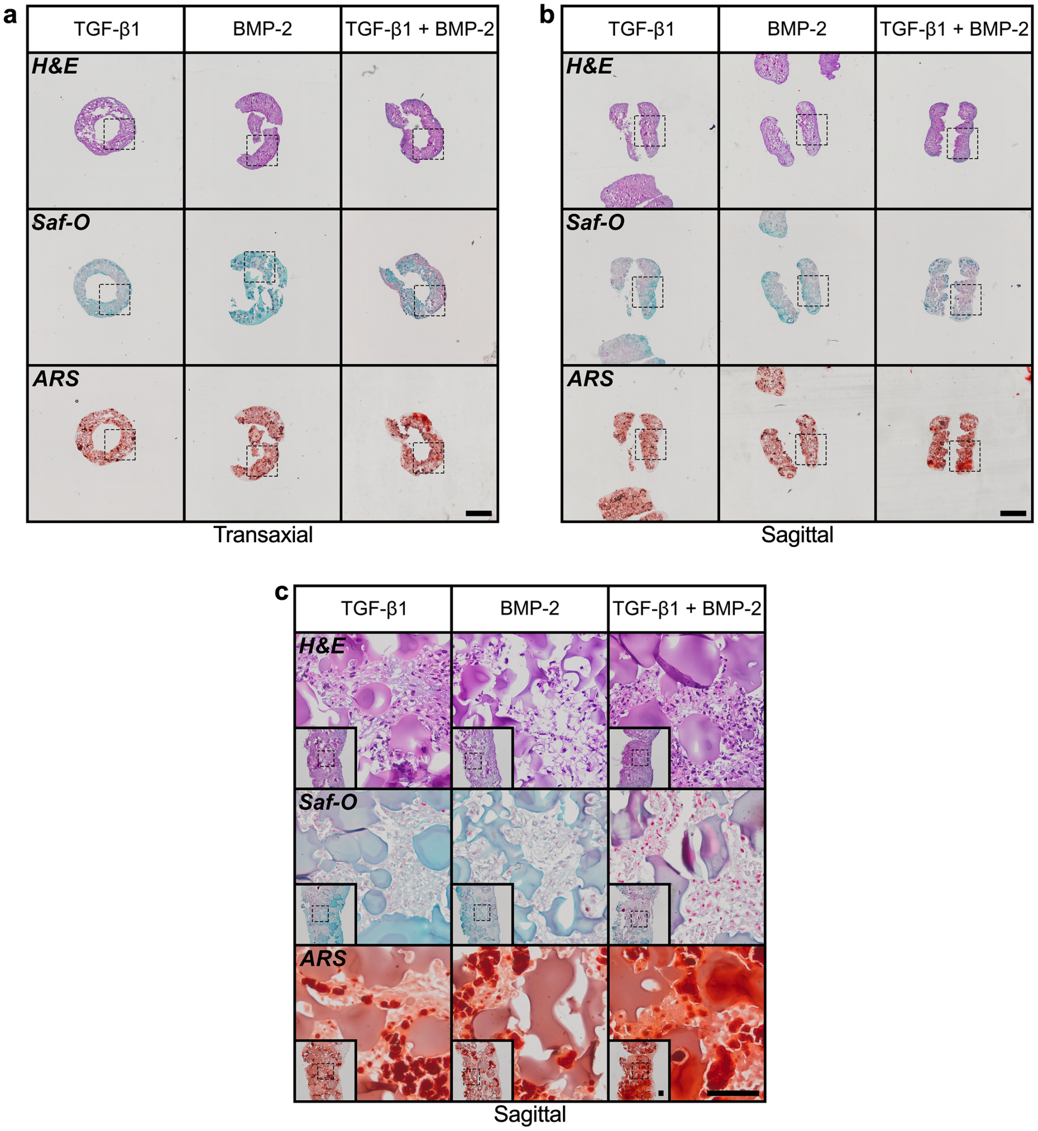

Fig. S2. *In vitro* histological evaluation of engineered hMSC condensate tube early chondrogenic priming. (a,b) Representative histological Hematoxylin & Eosin (H&E), Safranin-O/Fast green (Saf-O), and Alizarin Red S (ARS) staining of transaxial and sagittal sections of hMSC tubes containing TGF-β1-loaded, BMP-2-loaded, or TGF-β1 + BMP-2-loaded microparticles at day 8. Scale bars, 1 mm (dotted squares show areas used in 10x images in Fig. 1F or S2C). (c) Representative H&E, Saf-O, and ARS staining of sagittal hMSC tube sections. Scale bars, 100 μm (dotted squares in insets show region of interest in high magnification image).

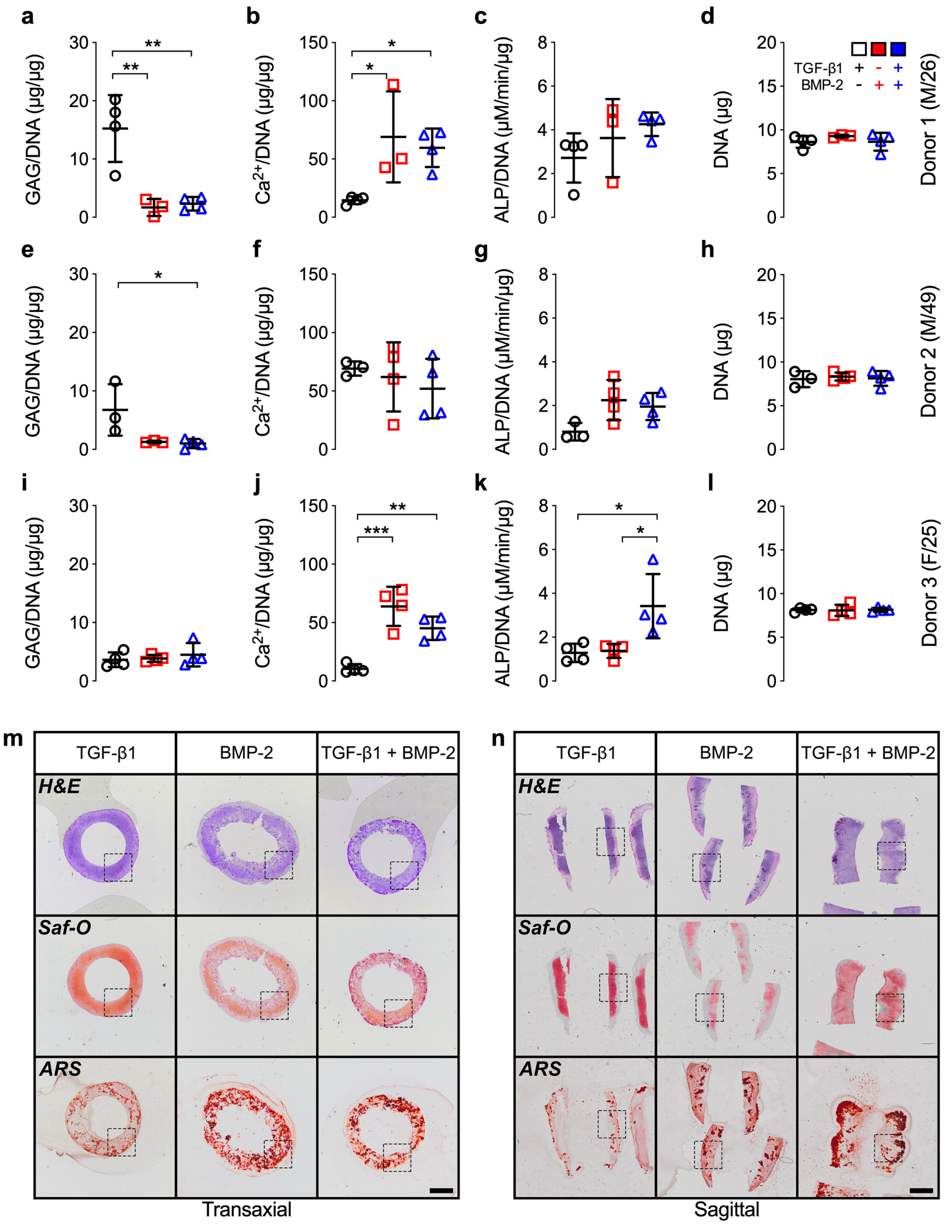

Fig. S3. *In vitro* biochemical and histological evaluation of engineered hMSC condensate tube maturation. Quantification of (a,e,i) GAG/DNA content, (b,f,j) Ca^{2+}/DNA content, (c,g,k) ALP activity/DNA, and (d,h,l) DNA content in hMSC tubes, from three separate donors, containing TGF-β1-loaded, BMP-2-loaded, or TGF-β1 + BMP-2-loaded microparticles at week 5 (donor 1: n = 4 (TGF-β1; TGF-β1 + BMP-2) or 3 (BMP-2), donor 2: n = 3 (TGF-β) or 4 (BMP-2; TGF-β1 + BMP-2), and donor 3: n = 4 (all groups) biologically independent samples per group; *$p<0.05$, **$p<0.01$, ***$p<0.001$; black circles = TGF-β1; red squares = BMP-2; blue triangles = TGF-β1+BMP-2). (m,n) Representative histological Hematoxylin & Eosin (H&E), Safranin-O/Fast green (Saf-O), and Alizarin Red S (ARS) staining of transaxial and sagittal hMSC tube sections. Scale bars, 1 mm (dotted squares show areas used in 10x images in Fig. 2K,L). Individual data points shown with mean ± SD. Analyzed by one-way ANOVA with Tukey's *post hoc* test ($p<0.05$ or lower considered significant).

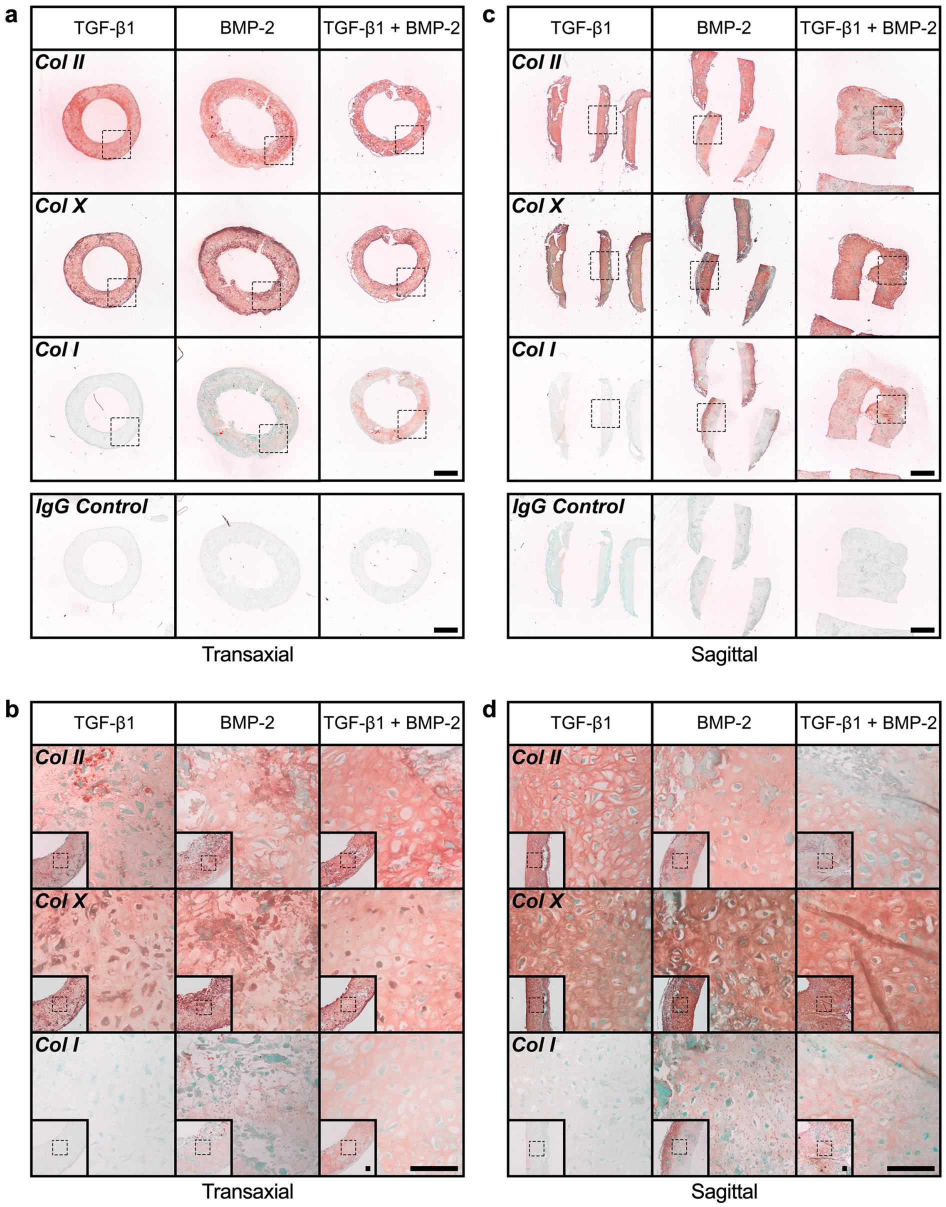

Fig. S4. *In vitro* immunohistochemical evaluation of engineered hMSC condensate tube maturation. (a,c) Representative immunohistochemical collagen (Col) II, Col X, and Col I staining of transaxial and sagittal sections of hMSC tubes containing TGF-β1-loaded, BMP-2-loaded, or TGF-β1 + BMP-2-loaded microparticles at week 5, with representative IgG negative controls. Scale bars, 1 mm (dotted squares show areas used in 10x images in Fig. S4B,D). (b,d) Representative immunohistochemical Col II, Col X, and Col I staining of transaxial and sagittal hMSC tube sections. Scale bars, 100 μm (dotted squares in insets show region of interest in high magnification image).

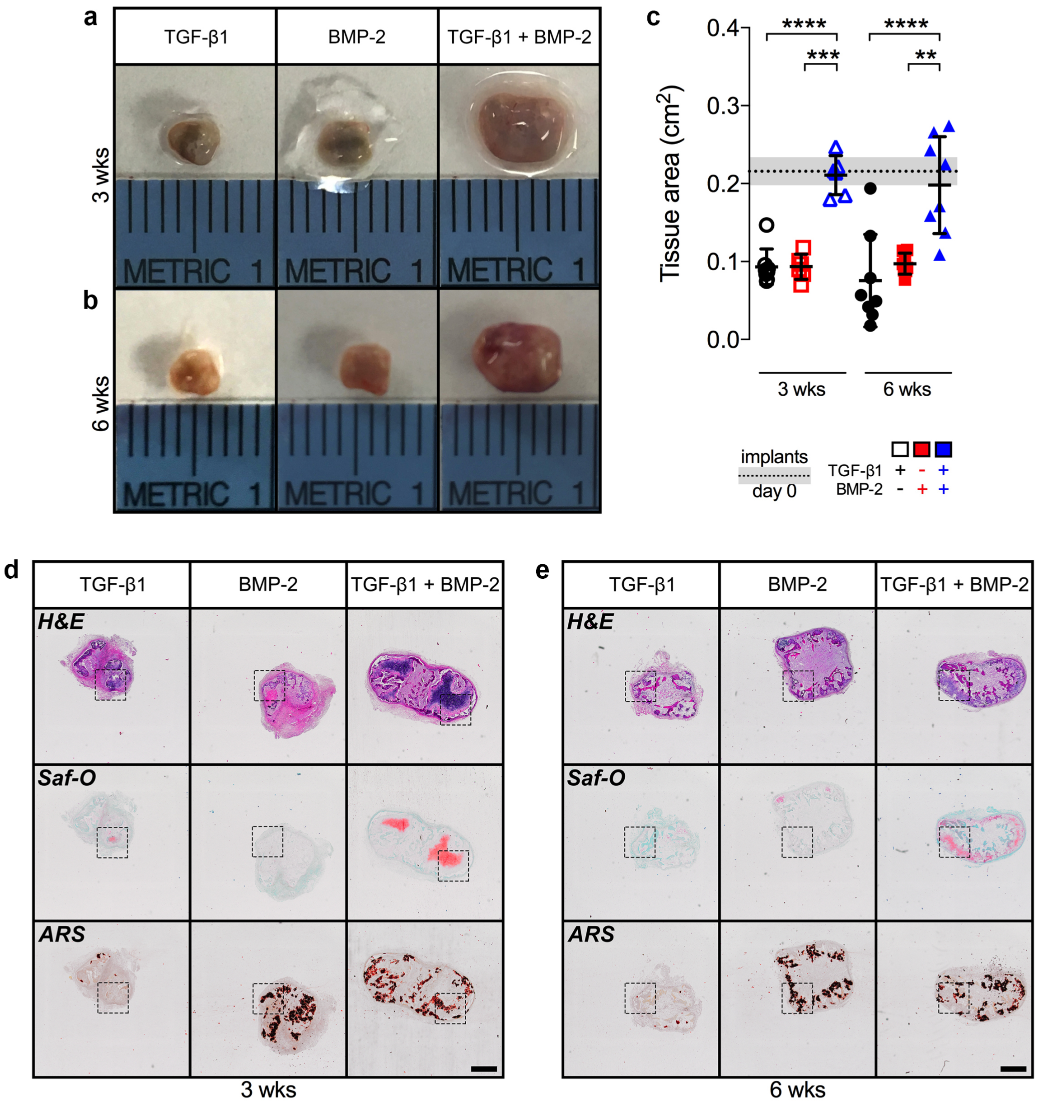

Fig. S5. ***Ex vivo*** **macroscopic and histological evaluation of subcutaneous bone tissue induced by engineered hMSC condensate tubes.** (a,b) Representative gross macroscopic images of hMSC tube explants containing TGF-β1-loaded, BMP-2-loaded, or TGF-β1 + BMP-2-loaded microparticles at week 3 and 6, selected based on mean tissue area. (c) hMSC explant tissue area quantification, shown with mean ± SD (gray shading) construct size across groups at the time of implantation (3 wks: n = 8 (TGF-β1) or 6 (BMP-2; TGF-β1 + BMP-2), and 6 wks: n = 8 (all groups) biologically independent samples per group; **$p<0.01$, ***$p<0.001$, ****$p<0.0001$; black circles = TGF-β1; red squares = BMP-2; blue triangles = TGF-β1+BMP-2; open symbols = 3 wks; closed symbols = 6 wks). (d,e) Representative histological Hematoxylin & Eosin (H&E), Safranin-O/Fast green (Saf-O), and Alizarin Red S (ARS) staining of sagittal hMSC tube explant sections at week 3 and 6. Scale bars, 1 mm (dotted squares show areas used in 10x images in Fig. 5a,b). Individual data points shown with mean ± SD. Analyzed by two-way ANOVA with Tukey's *post hoc* test ($p<0.05$ or lower considered significant).

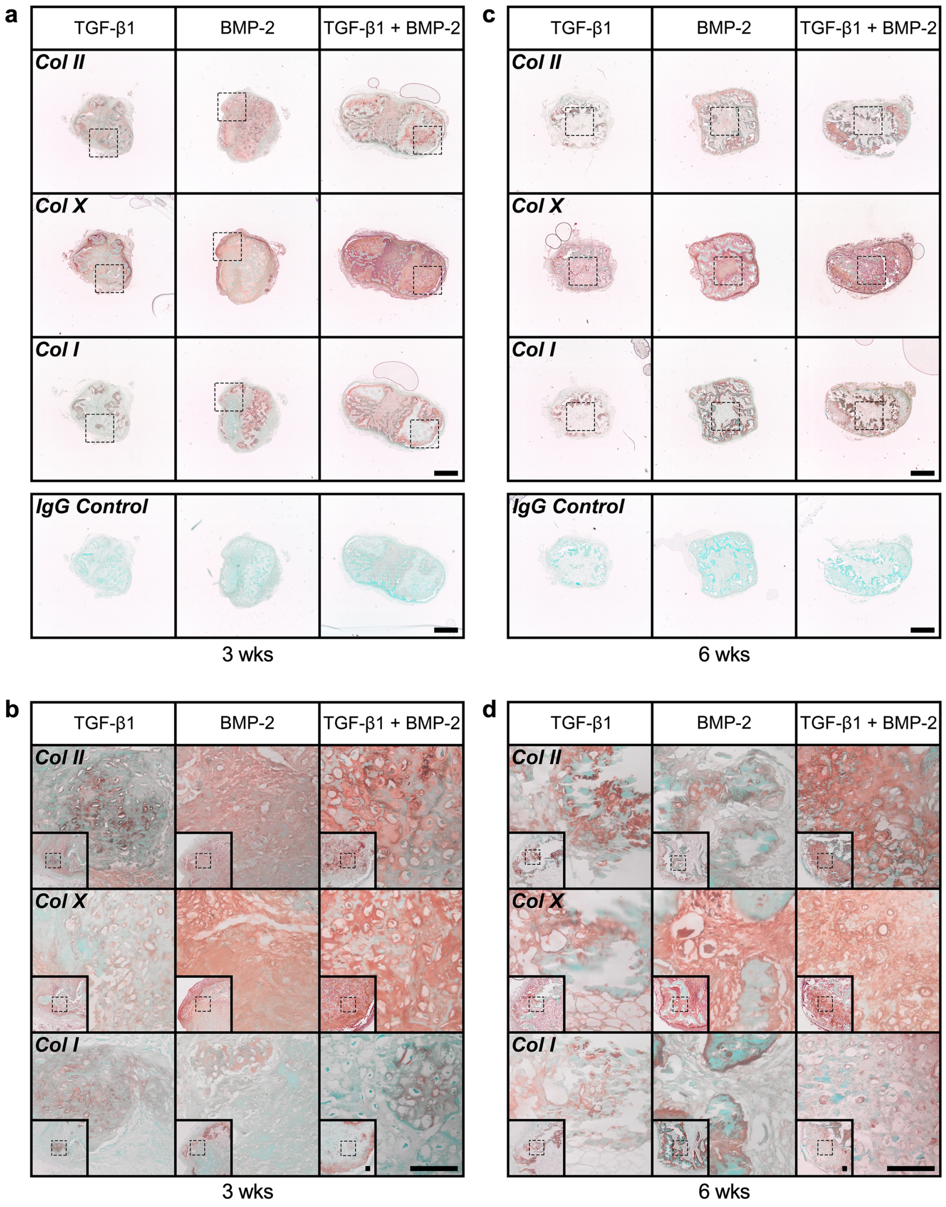

Fig. S6. *Ex vivo* immunohistochemical evaluation of subcutaneous bone tissue induced by engineered hMSC condensate tubes. (a,c) Representative immunohistochemical collagen (Col) II, Col X, and Col I staining of transaxial sections of hMSC tube explants containing TGF-β1-loaded, BMP-2-loaded, or TGF-β1 + BMP-2-loaded microparticles at week 3 and 6, with representative IgG negative controls. Scale bars, 1 mm (dotted squares show areas used in 10x images in Fig. S6b,d). (b,d) Representative immunohistochemical Col II, Col X, and Col I staining of sagittal hMSC tube explant sections at week 3 and 6. Scale bars, 100 μm (dotted squares in insets show region of interest in high magnification image).

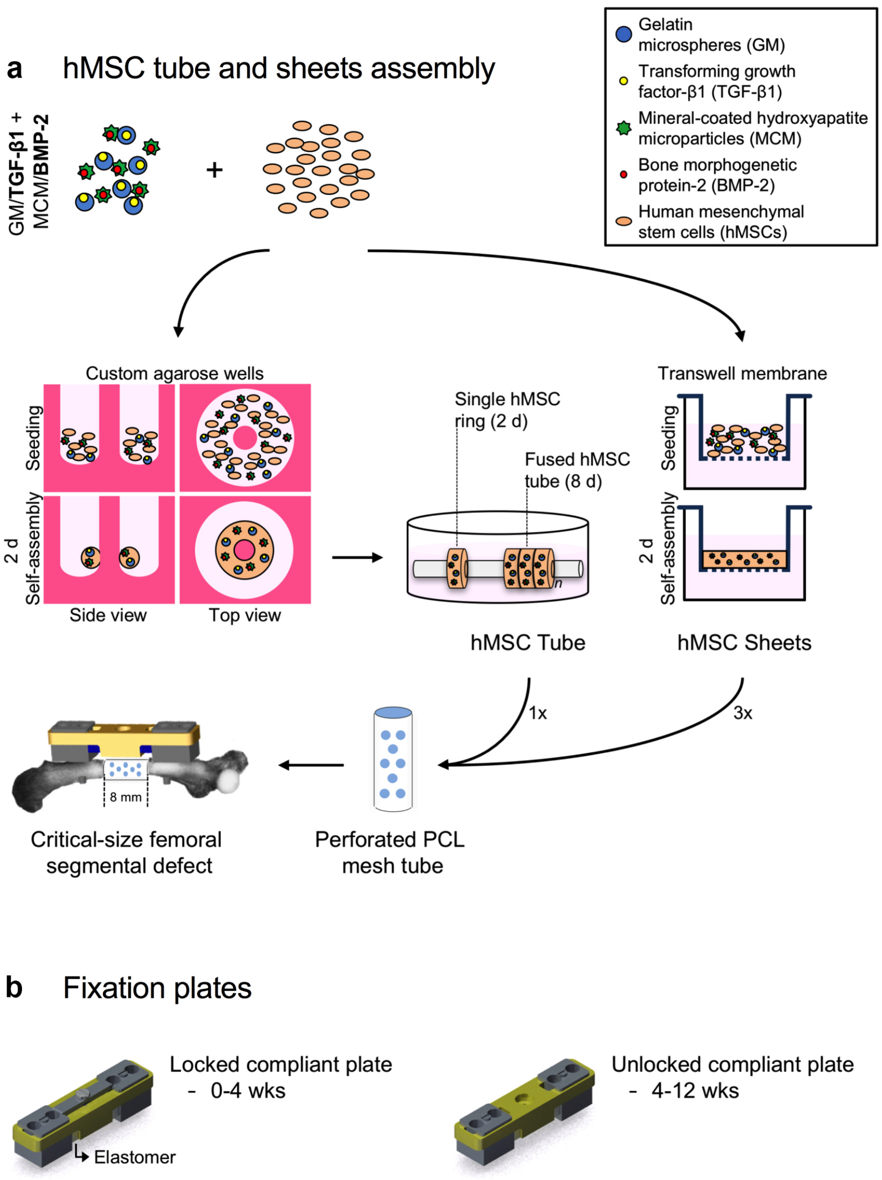

Fig. S7. Schematic of hMSC condensate tube and sheet assembly for femoral defect implantation. (a) hMSCs were mixed with TGF-β1-loaded gelatin microspheres and BMP-2-loaded mineral-coated hydroxyapatite microparticles [TGF-β1 + BMP-2], seeded in custom agarose culture wells and allowed to self-assemble into hMSC rings for 2 days before fusion into tubes by 8 days, or seeded onto membranes of transwell inserts and allowed to self-assemble into hMSC sheets for 2 days. One tube or three sheets (for identical cell number, microparticle concentration, and morphogen dose) were loaded into perforated polycaprolactone (PCL) nanofiber mesh tubes and implanted in critical-sized rat femoral segmental defects. (b) Limbs were stabilized with custom compliant fixation plates that were initially implanted in a locked configuration (0-4 weeks) to prevent load transfer, but were unlocked at week 4 to initiate ambulatory load transfer (4-12 weeks). The bone with fixation plate in (a) and the schematic comparison of fixation plates were adapted from refs 34 and 35 with permission from AAAS and the authors.

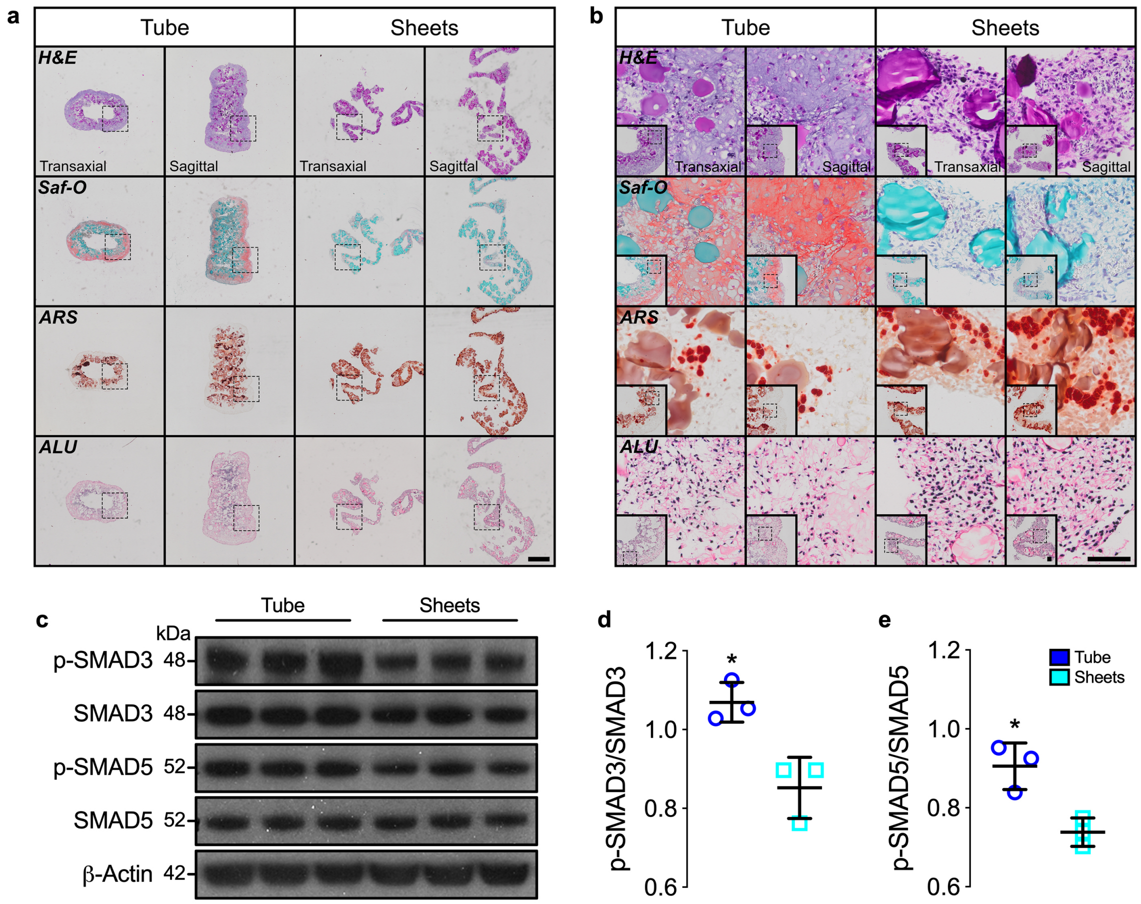

Fig. S8. *In vitro* histological and biochemical evaluation of engineered hMSC condensate tube and sheet implants. (a,b) Representative histological Hematoxylin & Eosin (H&E), Safranin-O/Fast green (Saf-O), and Alizarin Red S (ARS) staining, and *in situ* hybridization for human Alu repeats of transaxial and sagittal sections of hMSC tube and sheet implants containing TGF-β1 + BMP-2-loaded microparticles at day 8 and day 2, respectively. Scale bars, 1 mm ((a) dotted squares show areas used in 10x images in Fig. S8b) and 100 µm ((b) dotted squares in insets show region of interest in high magnification image). (c) Immunoblots and (d) relative quantification of p-SMAD3/SMAD3 and (e) p-SMAD5/SMAD5. β-Actin served as loading control (n = 3 biologically independent samples per group; *$p<0.05$; blue circles = hMSC tubes; cyan squares = hMSC sheets). Individual data points shown with mean ± SD. Analyzed by unpaired Student's *t*-test ($p<0.05$ or lower considered significant).

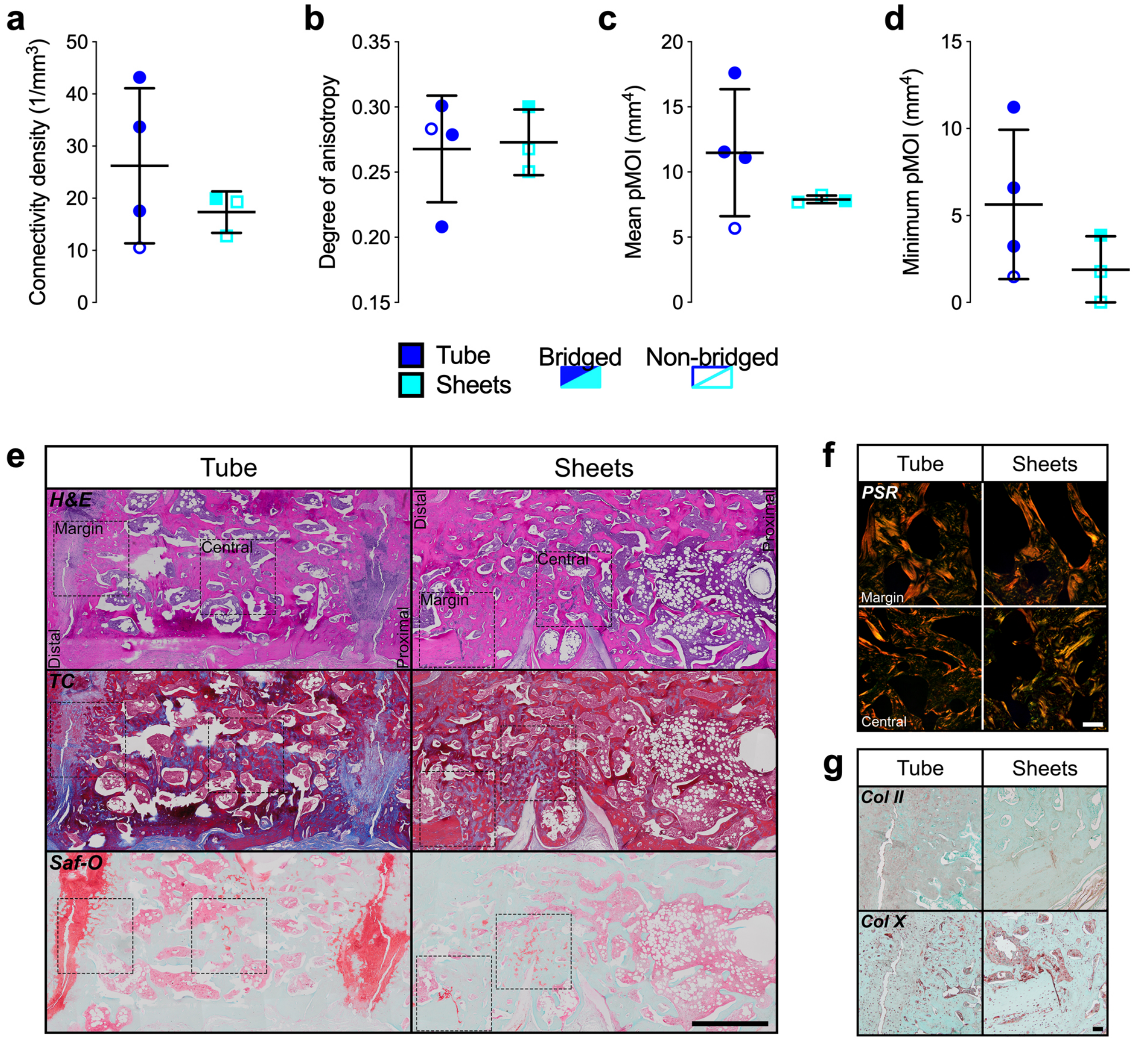

Fig. S9. *Ex vivo* microCT, histological, and immunohistochemical evaluation of femoral defect healing induced by engineered hMSC condensate tubes and sheets. Morphometric analysis of (a) connectivity density, (b) degree of anisotropy, (c) mean polar moment of inertia (pMOI), and (d) minimum pMOI in defects implanted with hMSC tubes or sheets containing TGF-β1 + BMP-2-loaded microparticles at week 12 (n = 4 (tubes) or 3 (sheets) biologically independent samples per group; blue circles = hMSC tubes; cyan squares = hMSC sheets; open symbols = non-bridged; closed symbols = bridged). (e) Representative histological Hematoxylin & Eosin (H&E), Masson's Trichrome (TC), and Safranin-O/Fast green (Saf-O) staining of sagittal defect explant sections showing the complete defect; images oriented distal-to-proximal from left-to-right. (f) Polarized light microscopy of picrosirius red-stained histological sections (defect margin and center). (g) Representative immunohistochemical collagen (Col) II and Col X staining of sagittal hMSC defect explant sections showing the defect margin. Scale bars, 2 mm ((e) dotted squares show areas used in 10x images in Fig. 7h) and 100 µm ((f)). Individual data points shown with mean ± SD. Analyzed by unpaired Student's *t*-test ($p<0.05$ or lower considered significant).

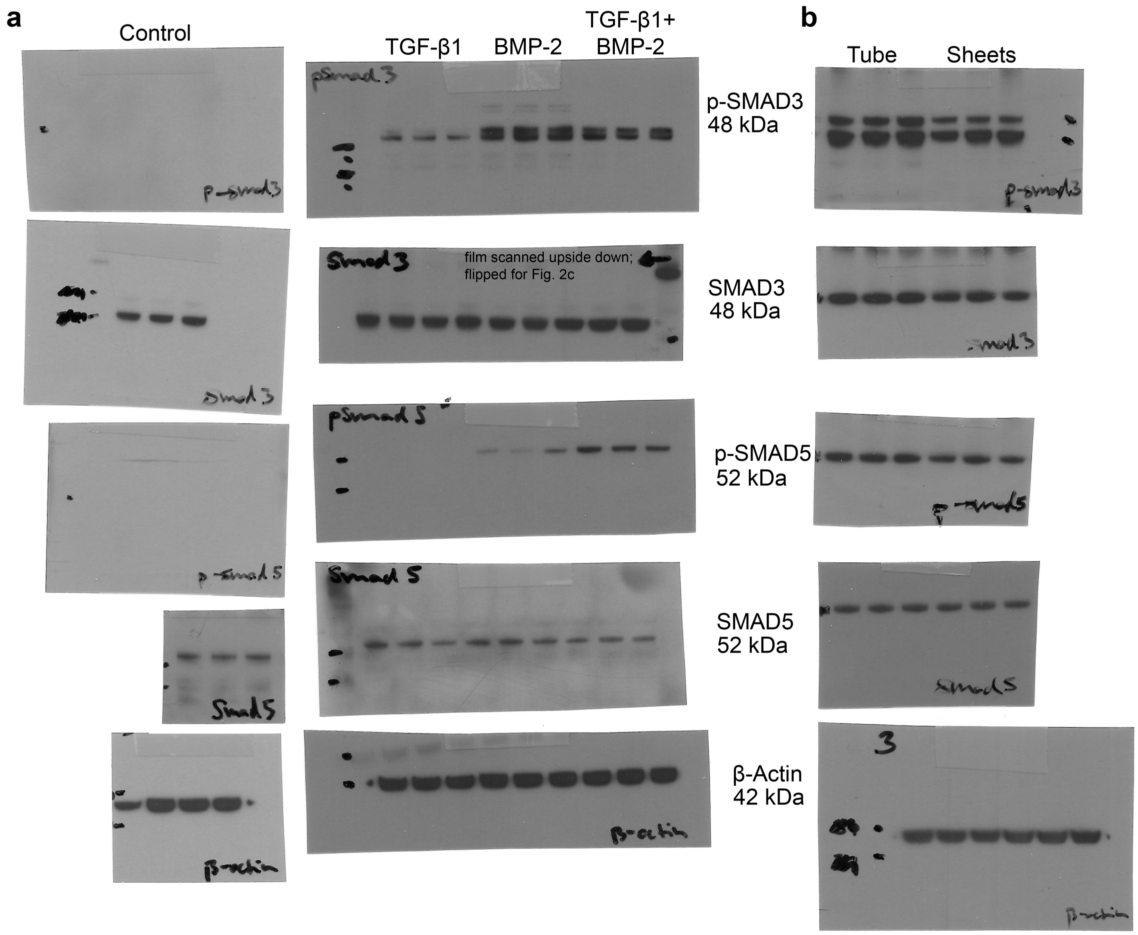

Fig. S10. Raw uncropped immunoblot images to support *in vitro* biochemical evaluation of (a) engineered hMSC condensate tube early chondrogenic priming in Fig. 2c, and (b) engineered hMSC condensate tube and sheet implants in Fig. S8c.
