# Supplementary material for: Scaffold-free human mesenchymal stem cell construct geometry regulates long bone regeneration
Source: Commun Biol. 2021 Jan 19;4:89. doi: 10.1038/s42003-020-01576-y (PMC7815708; doi:10.1038/s42003-020-01576-y)
Supplement: Supplementary file 2 — Description of Additional Supplementary Files [file 42003_2020_1576_MOESM2_ESM.pdf]

Description of Additional Supplementary Files

File Name: Supplementary Movie 1
Description: Ex vivo micro CT evaluation of mouse subcutaneous bone induced by engineered hMSC condensate tubes by week 3.Representative 3-D micro CT reconstructions (rotating around x-axis) of hMSC tube explants containing TGF-β1-loaded (left), BMP-2-loaded (middle), or TGF-β1 + BMP-2-loaded (right) microparticles, selected based on mean bone volume

File Name: Supplementary Movie 2
Description: Ex vivo micro CT evaluation of mouse subcutaneous bone induced by engineered hMSC condensate tubes by week 6.Representative 3-D microCT reconstructions (rotating around x-axis) of hMSC tube explants containing TGF-β1-loaded (left), BMP-2-loaded (middle), or TGF-β1 + BMP-2-loaded (right) microparticles, selected based on mean bone volume.

File Name: Supplementary Movie 3
Description: Ex vivo microCT evaluation of rat femoral defect healing induced by engineered hMSC condensate tubes and sheets by week 12. Representative 3-D microCT defect reconstructions (rotating around x-axis), implanted with hMSC tubes (left) or sheets (right) containing TGF-β1 + BMP-2-loaded microparticles, selected based on mean bone volume, with virtual transaxial sectioning until mid-shaft.

File Name: Supplementary Data 1
Description: Source data underlying all Figures and Supplementary Figures.
