# Supplementary material for: Scaffold-free human mesenchymal stem cell construct geometry regulates long bone regeneration
Source: Commun Biol. 2021 Jan 19;4:89. doi: 10.1038/s42003-020-01576-y (PMC7815708; doi:10.1038/s42003-020-01576-y)
Supplement: Supplementary file 6 — Supplementary Data 1 [file 42003_2020_1576_MOESM6_ESM.pdf]

Figure 2
Panel b **qPCR**

	SOX9			ACAN			COL2A1			ALP			RUNX2			COL1A1		
**TGFb1**	7.96	7.22	7.00	17.33	8.19	10.82	468.75	164.86	324.69	2.12	0.65	1.16	1.41	0.92	1.11	3.75	5.29	6.25
**BMP-2**	21.70	21.49	14.54	62.23	42.49	39.49	372.71	914.63	1161.58	32.07	47.38	64.72	1.34	1.72	1.41	5.13	4.77	5.14
**TGF-b1+BMP-2**	29.39	33.85	54.12	74.52	134.56	130.90	1911.58	1333.07	1520.57	161.64	316.71	384.39	1.36	2.25	2.61	2.05	7.00	8.10

Panel d **Immunoblot Fold-change pSmad3/Smad3**

TGFb1	BMP-2	TGF-b1+BMP-2
72.00	252.14	284.99
59.93	244.66	267.32
60.67	280.84	193.36

Panel e **Immunoblot Fold-change pSmad5/Smad5**

TGFb1	BMP-2	TGF-b1+BMP-2
1.40	29.47	205.67
0.66	25.94	134.10
2.61	68.84	159.98

Figure 3

Panel b Macro Tube height

TGFb1	BMP-2	TGF-b1+BMP-2
2.52	2.44	2.47
2.28	2.40	2.39
2.48	2.37	2.50

Panel c Macro Tube width

TGFb1	BMP-2	TGF-b1+BMP-2
0.65	0.61	0.65
0.63	0.63	0.61
0.62	0.66	0.64

Panel f Biochem GAG/DNA

TGFb1			BMP-2			TGF-b1+BMP-2		
Mean	SD	n	Mean	SD	n	Mean	SD	n
8.70	6.54	11	2.44	1.46	10	2.61	1.97	12

Panel g Biochem Ca/DNA

TGFb1			BMP-2			TGF-b1+BMP-2		
Mean	SD	n	Mean	SD	n	Mean	SD	n
28.01	26.82	11	64.63	25.68	11	52.28	17.78	12

Panel h Biochem ALP/DNA

TGFb1			BMP-2			TGF-b1+BMP-2		
Mean	SD	n	Mean	SD	n	Mean	SD	n
1.68	1.09	11	2.31	1.34	11	3.21	1.32	12

Panel i Biochem DNA

TGFb1			BMP-2			TGF-b1+BMP-2		
Mean	SD	n	Mean	SD	n	Mean	SD	n
8.28	0.63	11	8.50	0.68	11	8.31	0.74	12

Panel j Biochem GAG

TGFb1			BMP-2			TGF-b1+BMP-2		
Mean	SD	n	Mean	SD	n	Mean	SD	n
72.91	57.66	11	20.19	11.58	10	23.38	14.50	11

Panel k Biochem Ca

TGFb1			BMP-2			TGF-b1+BMP-2		
Mean	SD	n	Mean	SD	n	Mean	SD	n
227.92	213.27	11	553.05	239.96	11	444.57	182.66	12

Panel l Biochem ALP

TGFb1			BMP-2			TGF-b1+BMP-2		
Mean	SD	n	Mean	SD	n	Mean	SD	n
14.08	9.53	11	19.92	12.56	11	27.04	11.95	12

Figure 4

Panel e Ex vivo microCT Bone volume

	TGFb1								BMP-2								TGF-b1+BMP-2							
**3 wks**	0.42	0.44	0.07	0.27	2.62	1.12	0.81	0.86	0.21	1.02	0.79	0.43	1.36	0.70			4.71	4.23	5.52	4.96	2.61	3.81		
**6 wks**	0.31	0.03	0.58	0.20	1.35	1.58	5.28	9.11	2.39	2.58	1.45	1.50	2.26	2.21	1.75	3.58	6.05	18.71	6.64	3.34	7.02	15.17	10.62	14.02

Panel f Ex vivo microCT Trabecular number

	TGFb1								BMP-2								TGF-b1+BMP-2							
**3 wks**	0.15	0.17	0.03	0.10	0.45	0.24	0.15	0.23	0.13	0.42	0.24	0.16	0.35	0.19			0.89	0.70	0.74	0.76	0.51	0.63		
**6 wks**	0.17	0.04	0.19	0.08	0.32	0.28	0.69	1.05	0.42	0.39	0.34	0.38	0.30	0.65	0.36	0.49	0.89	1.67	0.77	0.51	0.80	1.64	0.96	1.07

Panel g Ex vivo microCT Trabecular thickness

	TGFb1								BMP-2								TGF-b1+BMP-2							
**3 wks**	0.06	0.06	0.06	0.07	0.09	0.09	0.09	0.09	0.05	0.06	0.08	0.07	0.08	0.08			0.07	0.07	0.08	0.07	0.07	0.07		
**6 wks**	0.06	0.05	0.08	0.08	0.12	0.12	0.10	0.10	0.11	0.11	0.11	0.11	0.12	0.11	0.11	0.12	0.09	0.09	0.12	0.11	0.11	0.10	0.12	0.11

Panel h Ex vivo microCT Trabecular separation

	TGFb1								BMP-2								TGF-b1+BMP-2							
**3 wks**	1.35	1.18	1.43	1.42	1.06	1.47	1.61	1.28	1.10	1.01	1.34	1.38	1.34	1.47			0.85	0.94	1.05	0.88	1.15	1.02		
**6 wks**	1.39	0.73	1.44	1.49	1.22	1.32	1.04	0.84	1.11	1.24	1.34	1.08	1.21	0.75	1.13	1.25	0.86	0.64	0.94	1.11	0.91	0.55	0.97	0.85

Figure 6

Panel a In vivo microCT Bone volume

	Tube			Sheets		
	Mean	SD	n	Mean	SD	n
0 wks	0.00	0.00	4	0.00	0.00	3
4 wks	16.40	1.35	4	15.54	3.09	3
8 wks	29.77	12.38	4	24.70	2.99	3
12 wks	38.60	13.42	4	27.89	2.87	3

Panel b In vivo microCT Bone accumulation rate

	TGFb1				BMP-2		
4 wks	4.16	4.52	4.03	3.70	3.42	4.78	3.46
8 wks	7.64	4.45	3.15	2.54	2.14	0.87	2.86
12 wks	2.88	3.06	0.93	1.97	2.12	1.65	1.88

Panel c In vivo microCT Bridging

	Tube		Sheets	
	Mean	n	Mean	n
0 wks	0.00	4	0.00	3
4 wks	0.00	4	0.00	3
8 wks	50.00	4	33.30	3
12 wks	75.00	4	33.30	3

Figure 7

Panel d **Ex vivo microCT** **Trabecular number**

Tube	Sheets
1.73	1.19
1.01	0.78
0.64	0.80
1.32	

Panel e **Ex vivo microCT** **Trabecular thickness**

Tube	Sheets
0.20	0.21
0.26	0.27
0.24	0.20
0.20	

Panel f **Ex vivo microCT** **Trabecular separation**

Tube	Sheets
0.76	1.19
0.87	1.09
1.25	1.19
0.73	

Panel g **Ex vivo microCT** **Ectopic bone**

Tube	Sheets
4.48	5.30
6.27	5.11
4.41	14.03
5.75	

Figure 8

Panel b **Histomorphometry** **Bone area fraction**

Tube	Sheets
60.78	29.44
36.19	31.75
49.76	31.49
57.98	
