# Supplementary material for: Scaffold-free human mesenchymal stem cell construct geometry regulates long bone regeneration
Source: Commun Biol. 2021 Jan 19;4:89. doi: 10.1038/s42003-020-01576-y (PMC7815708; doi:10.1038/s42003-020-01576-y)
Supplement: Supplementary file 7 — Reporting Summary [file 42003_2020_1576_MOESM7_ESM.pdf]

nature research

Corresponding author(s): Eben Alsberg

Last updated by author(s): Dec 9, 2019

Reporting Summary

Nature Research wishes to improve the reproducibility of the work that we publish. This form provides structure for consistency and transparency in reporting. For further information on Nature Research policies, see Authors & Referees and the Editorial Policy Checklist .

Statistics

For all statistical analyses, confirm that the following items are present in the figure legend, table legend, main text, or Methods section.

n/a	Confirmed	
☐	☒	The exact sample size (n) for each experimental group/condition, given as a discrete number and unit of measurement
☐	☒	A statement on whether measurements were taken from distinct samples or whether the same sample was measured repeatedly
☐	☒	The statistical test(s) used AND whether they are one- or two-sided<br>*Only common tests should be described solely by name; describe more complex techniques in the Methods section.*
☒	☐	A description of all covariates tested
☐	☒	A description of any assumptions or corrections, such as tests of normality and adjustment for multiple comparisons
☐	☒	A full description of the statistical parameters including central tendency (e.g. means) or other basic estimates (e.g. regression coefficient) AND variation (e.g. standard deviation) or associated estimates of uncertainty (e.g. confidence intervals)
☒	☐	For null hypothesis testing, the test statistic (e.g. F, t, r) with confidence intervals, effect sizes, degrees of freedom and P value noted<br>*Give P values as exact values whenever suitable.*
☒	☐	For Bayesian analysis, information on the choice of priors and Markov chain Monte Carlo settings
☒	☐	For hierarchical and complex designs, identification of the appropriate level for tests and full reporting of outcomes
☒	☐	Estimates of effect sizes (e.g. Cohen's d, Pearson's r), indicating how they were calculated

Our web collection on statistics for biologists contains articles on many of the points above.

Software and code

Policy information about availability of computer code

Data collection	No software was used.
Data analysis	No software was used.

For manuscripts utilizing custom algorithms or software that are central to the research but not yet described in published literature, software must be made available to editors/reviewers. We strongly encourage code deposition in a community repository (e.g. GitHub). See the Nature Research guidelines for submitting code & software for further information.

Data

Policy information about availability of data

All manuscripts must include a data availability statement. This statement should provide the following information, where applicable:

- Accession codes, unique identifiers, or web links for publicly available datasets
- A list of figures that have associated raw data
- A description of any restrictions on data availability

All data needed to evaluate the conclusions in the paper are present in the paper and/or the Supplementary Materials.

Field-specific reporting

Please select the one below that is the best fit for your research. If you are not sure, read the appropriate sections before making your selection.

☒ Life sciences ☐ Behavioural & social sciences ☐ Ecological, evolutionary & environmental sciences

For a reference copy of the document with all sections, see nature.com/documents/nr-reporting-summary-flat.pdf

Life sciences study design

All studies must disclose on these points even when the disclosure is negative.

Sample size	Sample sizes were chosen based on previous studies from our group.
Data exclusions	No data were excluded from the analyses.
Replication	All attempts at replication were successful.
Randomization	Ectopic: Two constructs from the same group were on the same side in each animal, which were evenly matched with other groups among animals for homogeneous pairing distribution. Orthotopic: Treatment groups were randomly allocated.
Blinding	Investigators were blinded to group allocation during data collection and analyses.

Reporting for specific materials, systems and methods

We require information from authors about some types of materials, experimental systems and methods used in many studies. Here, indicate whether each material, system or method listed is relevant to your study. If you are not sure if a list item applies to your research, read the appropriate section before selecting a response.

Materials & experimental systems

n/a	Involved in the study	
☐	☒	Antibodies
☒	☐	Eukaryotic cell lines
☒	☐	Palaeontology
☐	☒	Animals and other organisms
☒	☐	Human research participants
☒	☐	Clinical data

Methods

n/a	Involved in the study	
☒	☐	ChIP-seq
☒	☐	Flow cytometry
☒	☐	MRI-based neuroimaging

Antibodies

Antibodies used	Abcam: anti-phospho-SMAD3 [ab52903]; anti-phospho-SMAD5 [ab92698]; anti-SMAD3 [ab40854]; anti-SMAD5 [ab40771]; anti-Col I [ab138492], anti-Col II [ab34712], and anti-Col X [ab58632]. Sigma-Aldrich: anti-β-Actin [A1978].
Validation	Antibodies were used according to the manufacturers' specifications. Rabbit IgG (Vector Laboratories) served as negative control for immunostaining.

Animals and other organisms

Policy information about studies involving animals; ARRIVE guidelines recommended for reporting animal research

Laboratory animals	Ectopic: 9-week-old male NCr nude mice were obtained from the Athymic Animal and Xenograft Core Facility at Case Western Reserve University. Orthotopic: 12-week-old male Rowett nude rats were obtained from Taconic Biosciences Inc.
Wild animals	Study did not involve wild animals.
Field-collected samples	Study did not involve samples collected from the field.
Ethics oversight	All procedures were performed in strict accordance with the NIH Guide for the Care and Use of Laboratory Animals, and the policies of the Case Western Reserve University Institutional Animal Care and Use Committee (Ectopic: Protocol No. 2014-0096; Orthotopic: Protocol No. 2015-0081).

Note that full information on the approval of the study protocol must also be provided in the manuscript.
